# Pre-capture multiplexing provides additional power to detect copy number variation in exome sequencing

**DOI:** 10.1186/s12859-021-04246-w

**Published:** 2021-07-20

**Authors:** Dayne L. Filer, Fengshen Kuo, Alicia T. Brandt, Christian R. Tilley, Piotr A. Mieczkowski, Jonathan S. Berg, Kimberly Robasky, Yun Li, Chris Bizon, Jeffery L. Tilson, Bradford C. Powell, Darius M. Bost, Clark D. Jeffries, Kirk C. Wilhelmsen

**Affiliations:** 1 Department of Genetics, UNC School of Medicine, Chapel Hill, USA; 2 Renaissance Computing Institute, Chapel Hill, USA; 3 UNC School of Information and Library Science, Chapel Hill, USA; 4 Department of Biostatistics, UNC Gillings School of Global Public Health, Chapel Hill, USA; 5 Department of Neurology, UNC School of Medicine, Chapel Hill, USA

**Keywords:** Exome sequencing, Copy number variation, Capture

## Abstract

**Background:**

As exome sequencing (ES) integrates into clinical practice, we should make every effort to utilize all information generated. Copy-number variation can lead to Mendelian disorders, but small copy-number variants (CNVs) often get overlooked or obscured by under-powered data collection. Many groups have developed methodology for detecting CNVs from ES, but existing methods often perform poorly for small CNVs and rely on large numbers of samples not always available to clinical laboratories. Furthermore, methods often rely on Bayesian approaches requiring user-defined priors in the setting of insufficient prior knowledge. This report first demonstrates the benefit of multiplexed exome capture (pooling samples prior to capture), then presents a novel detection algorithm, mcCNV (“multiplexed capture CNV”), built around multiplexed capture.

**Results:**

We demonstrate: (1) multiplexed capture reduces inter-sample variance; (2) our mcCNV method, a novel depth-based algorithm for detecting CNVs from multiplexed capture ES data, improves the detection of small CNVs. We contrast our novel approach, agnostic to prior information, with the the commonly-used ExomeDepth. In a simulation study mcCNV demonstrated a favorable false discovery rate (FDR). When compared to calls made from matched genome sequencing, we find the mcCNV algorithm performs comparably to ExomeDepth.

**Conclusion:**

Implementing multiplexed capture increases power to detect single-exon CNVs. The novel mcCNV algorithm may provide a more favorable FDR than ExomeDepth. The greatest benefits of our approach derive from (1) not requiring a database of reference samples and (2) not requiring prior information about the prevalance or size of variants.

**Supplementary Information:**

The online version contains supplementary material available at 10.1186/s12859-021-04246-w.

## Background

In human genetics, individuals normally have two copies of each locus in the genome (one inherited from each parent). Deviations from the normal diploid state, known broadly as copy number variation, can cause phenotypic changes and Mendelian disorders. Technologies, e.g. microarray, exist for reliably detecting large (greater than 100 kilobases) copy number variants (CNVs). Over the last decade, the availability short-read DNA sequencing compelled numerous efforts to identify and characterize smaller variants. Sequencing cost, data burden, and the problem of classifying intronic and non-coding variants have led to exome sequencing (ES) as the preferred clinical sequencing modality. ES analysis most often focuses on identifying pathogenic single-nucleotide variants and insertion/deletions. CNV analysis can provide modest improvement in diagnostic yield [1], but existing data/analysis lacks the power to detect exon-level variation [2, 3]. Poor detection power to date obscures the true diagnostic potential of small CNVs.

Current analytic methodologies adequately detect large CNVs, but require large sample sizes (dozens to hundreds) and lack resolution for intragenic exon-level variation [4–7]. The prevalence and clinical importance of exon-level CNVs remain largely unknown due to inadequate power in ES studies and limited access to clinical genome sequencing data. Recent work on a subset of 1507 genes suggests intragenic CNVs account for 1.9% of total variants but 9.8% of pathogenic variants [8]. Additionally, the authors demonstrated 627/2844 (22%) of identified CNVs spanned a single (598) or partial (29) exon [8].

Targeted sequencing requires capturing the desired loci (e.g. exons) using sequence-specific oligonucleotide baits. Even when carefully designed and balanced, the differential efficiency of baits leads to variable read-depth across the exome. The GC content and length of targeted fragments contribute to the observed variable read-depth [9]; most ES analysis platforms incorporate a correction for GC content and exon length [10]. The variable read-depth in ES precludes the single-sample window-smoothing approaches successfully applied in GS data [11], e.g. Control-FREEC [12], CONDEL [13], CNV_IFTV [14], CNVnator [15], ERDS [16]; therefore we must rely on comparative analysis for interrogating copy number. Comparative analysis requires a set of reference controls; we presume the reference controls do not have the same rare CNVs as the test subject and accept not identifying common CNVs.

Comparing multiple samples, each captured independently, compounds the variable read-depth problem. The capture probability for each exon correlates between samples but with high variability [4]. In other words, we can gain information from similarly captured samples, but independent captures introduce significant noise. ExomeDepth attempts to circumvent the capture-to-capture variation by identifying a subset of samples from a large pool with low inter-sample variability [4]. Alternatively, CoNIFER [5], XHMM [6], and CODEX [7] use a latent factor model with spectral value decomposition to remove systematic noise, presumably introduced by capture-to-capture variation. These methods generally require very large sample sizes and often still lack power for exon-level resolution (e.g. CODEX defines a “short” CNV as spanning five contiguous exons).

Herein, we divide our report broadly into two parts. First, we demonstrate multiplexing the capture across samples reduces inter-sample variance and provides an appropriate set of controls for ExomeDepth, thus increasing the power to detect CNVs. Second, we introduce our novel algorithm, mcCNV (“multiplexed capture CNV”), specifically designed to utilize multiplexed capture exome data for estimating exon-level variation without prior information.

## Results

### Multiplexed capture reduces inter-sample variance

ES requires using molecular baits to “capture” the exonic DNA fragments during the library preparation (before sequencing). To expedite results to patients and simplify the workflow, in our experience most laboratories (including, by personal communication, the authors of the manuscript demonstrating the cost-efficiency of multiplexed capture [17]) capture each sample individually. The capture efficiency varies with timing, temperature, and substrate concentrations, making identical capture reproduction impossible. Alternatively, one could multiplex (pool) samples before capture, capturing the pool of samples simultaneously. Here we profile the inter-sample variance of individual capture versus multiplexed capture.

A multinomial process provides a logical framework for modeling targeted capture, with each target represented by an individual outcome. We can estimate the multinomial probability simplex for an exome capture by dividing the observed counts at each exon by the total mapped reads for the exome. The Dirichlet distribution, the conjugate prior for the multinomial distribution, defines distributions of probability simplexes. The Dirichlet distribution is parameterized by $\varvec{\alpha } = \{\alpha _1, \alpha _2, \ldots , \alpha _n\}$, where the expected probability for outcome *i* ($i = 1, 2, \ldots , n$) is given by $\alpha _i/\alpha _0,~\alpha _0 = \sum \varvec{\alpha }$. If $\varvec{\pi }$ is a probability simplex drawn from a Dirichlet with parameter $\varvec{\alpha }$, then the variance of $\varvec{\pi }$ is inversely proportional to $\alpha _0$. Therefore, we can approximate the inter-sample variance by fitting the Dirichlet distribution to each pool and interrogating the mean $\alpha$.

**Fig. 1:**
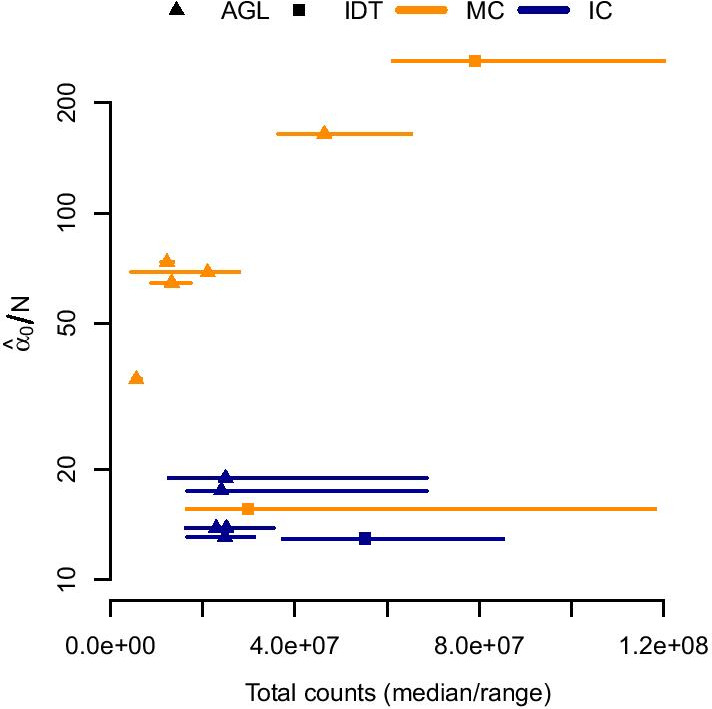
Multiplexed capture (MC) decreases variance with respect to independent captures (IC), as estimated by fitting the Dirichlet distribution. Total counts/sample given on the horizontal axis; mean $\alpha$ given on the vertical axis. $\alpha _0$ is inversely proportional to inter-sample variance. Each line/point represents a single pool. The point indicates the median total counts across the pool, with the range given by the line. Orange indicates a multiplexed capture; blue indicates independent captures. Triangles indicate pools using Agilent (AGL) capture; squares indicate Integrated DNA Technologies (IDT)

Using multiplexed capture, we sequenced three 16-sample pools and two 8-sample pools with Agilent baits and two 16-sample pools with IDT baits (Table 1). To compare to individually-captured Agilent data, we randomly selected 5 16-sample pools from the NCGENES cohort. We subset to exons with at least 5 and no greater than 2000 counts across all samples within a pool for numeric stability. We then used a Newton-Raphson algorithm [18] to fit the Dirichlet distribution to each pool; all pools converged to stable estimates. With one exception, we found multiplexed capture pools had greater $\alpha _0$ than their independently-captured counterparts (Fig. 1).

**Fig. 2:**
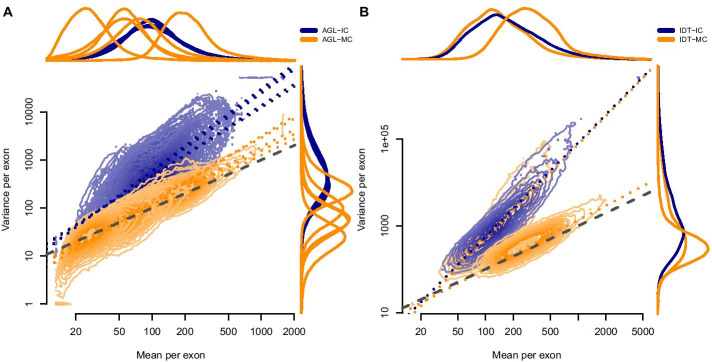
Mean-variance relationship demonstrates less dispersion in multiplexed capture. **a** Agilent (AGL) capture pools; **b** integrated DNA Technologies (IDT) capture pools. Mean counts per exon given on the horizontal axis; mean variance per exon given on the vertical axis. Contours show the distribution of points by pool. Dotted lines show the ordinary least squares regression fit. Orange indicates multiplexed capture pools; blue indicates independently captured pools. The dashed gray line represents the 1:1 relationship expected under a Poisson process. Lines above the plot show the density of mean values by pool; lines to the right of the plot show the density of variance values by pool

**Table 1:** Summary of whole-exome sequencing. ‘pool’ indicates the name of the pool of samples; ‘capture’ indicates the capture platform for the pool; ‘N’ gives the number of samples in the pool; ‘medExon’ gives the pool median of the subject median mapped molecule count per exon; ‘medTotal’ gives the median by pool of total mapped molecule counts per subject; ‘minTotal’ and ‘maxTotal’ give the minimum and maximum total mapped molecules; ‘rsdTotal’ gives the relative standard deviation (SD/mean*100) of total mapped molecules

Pool	Capture	N	medExon	medTotal	minTotal	maxTotal	rsdTotal
IDT-IC^a^	IDT	16	143	55,149,058	37,453,015	85,138,915	22.4
IDT-MC	IDT	16	93	29,772,684	16,674,468	118,147,912	64.2
IDT-RR	IDT	16	272	79,079,629	61,289,322	120,147,888	22.9
NCGENES^a^	Agilent	112	93	24,451,245	12,749,793	68,565,471	27.6
Pool1	Agilent	16	56	13,265,614	8,911,132	17,324,903	18.5
Pool2	Agilent	16	86	21,076,056	4,585,195	27,846,146	27.6
SMA1	Agilent	8	56	12,256,002	11,051,840	13,600,697	6.2
SMA2	Agilent	8	25	5,622,040	4,904,000	6,545,360	10.4
WGS	Agilent	16	196	46,406,224	36,496,097	65,200,410	16.4

^a^Indicates captures were performed independently on each sample within the pool, otherwise captures were multiplexed across all samples within the pool

**Table 2:** Number of CNV calls by subject and algorithm for the ‘WGS’ pool

Subject	Total			Duplications			Deletions		
	MC	ED	WG	MC	ED	WG	MC	ED	WG
NCG_00012	90	106	143	61	73	121	29	33	22
NCG_00237	82	101	165	50	64	129	32	37	36
NCG_00525	68	74	151	30	33	110	38	41	41
NCG_00593	45	58	142	22	28	81	23	30	61
NCG_00676	66	78	112	38	46	92	28	32	20
NCG_00790	5156	2204	121	19	37	92	5137	2167	29
NCG_00819	68	76	134	30	41	100	38	35	34
NCG_00840	78	92	157	44	52	115	34	40	42
NCG_00851	1151	859	141	28	51	102	1123	808	39
NCG_00857	59	75	119	10	15	81	49	60	38
NCG_00976	46	58	114	25	37	93	21	21	21
NCG_01023	59	95	143	32	60	113	27	35	30
NCG_01043	73	94	128	40	64	105	33	30	23
NCG_01076	36	57	105	7	22	78	29	35	27
NCG_01077	135	157	230	103	121	184	32	36	46
NCG_01117	95	101	154	72	78	129	23	23	25

‘MC’ indicates the mcCNV algorithm; ‘ED’ indicates the ExomeDepth algorithm; ‘WG’ indicates the overlap of ERDS/cnvpytor calls from matched whole-genome sequencing. Exons with any overlap of the repetitive and low-complexity regions, as defined in the Trost et al. manuscript, omitted from analysis

The multiplexed pool without decreased inter-sample variance, IDT-MC, had a much larger spread in sequencing depth across the pool (Table 1, Fig. 1). Looking at the total mapped molecules, the IDT-MC pool had over double the relative standard deviation (64.2%) of any other pool. We hypothesized the absent reduction in variation stemmed from poor library balance during the multiplexing step. We subsequently captured a new pool using the same DNA input, IDT-RR, and found comparable reductions in inter-sample variance (the pool with the highest $\alpha _0$ in Fig. 1).

Examining the mean-variance relationship demonstrated the same inter-sample variance reduction suggested by the Dirichlet parameter estimates (Fig. 2). The Agilent pools (Fig. 2a) segregated cleanly, with less dispersion in the multiplexed capture pools. Again, we found no variance reduction for the IDT-MC pool, overlapping with the independently-captured IDT-IC pool (Fig. 2b). We did, however, observe near-complete reduction in dispersion for the better-balanced IDT-RR pool.

### Multiplexed capture provides controls for ExomeDepth

**Fig. 3:**
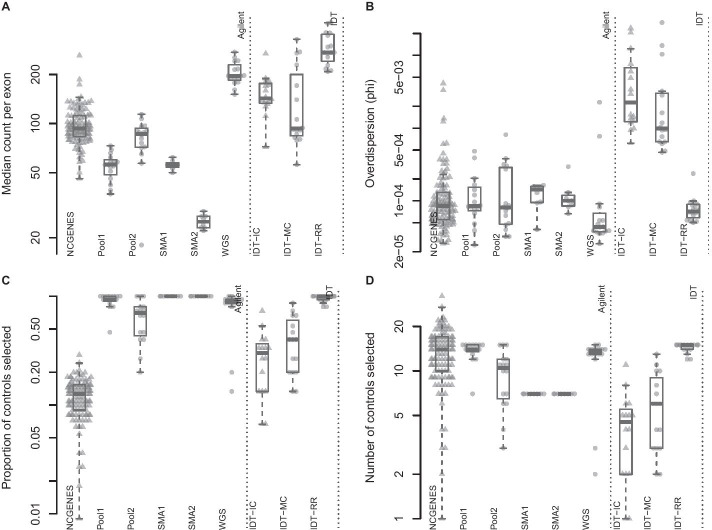
ExomeDepth control selection. **a** median count per exon; **b** estimated phi parameter from ExomeDepth; **c** proportion of available samples selected as a control; **d** total number of controls selected. Each point represents a single sample, with samples grouped by pool. Triangles indicate independently-captured samples; circles indiciate a single multiplexed capture within the pool. Dotted vertical line separates the two capture platforms

ExomeDepth requires a set of control subjects, summed into a reference vector of counts at each exon. ExomeDepth provides functionality to select appropriate controls from a set of subjects, often requiring hundreds of subjects to identify appropriate controls. Smaller research groups and clinical laboratories may struggle to build large databases of exomes, with the difficulty compounded by lot-to-lot variation and regular improvements to capture and sequencing chemistries. We wanted to know if the reduced inter-sample variance with multiplexed capture could provide an appropriate control set for ExomeDepth, eliminating the need for large databases of similarly-captured exomes. We found the reduced inter-sample variance with multiplexed capture leads to appropriate control selection for ExomeDepth (Fig. 3). Pool2, where we repeated the initial fragmentation five times, did not perform as well as the other multiplexed pools. We also found two samples within the WGS pool did not correlate well with the rest of the pool.

When we looked at independently-captured subjects, we found appropriate control sets for most of the 112 NCGENES subjects (Fig. 3d). However, ExomeDepth only selected 12.2% of available samples as controls, on average (Fig. 3c). Similarly, with the independently-captured IDT-IC pool we find low control numbers for most samples. While possible to select the same number of controls but exhibit differing dispersion, we observed little difference in the dispersion between independent and multiplexed capture (Fig. 3b). Overall, multiplexed capture provided appropriate controls for most samples tested and performed comparably to independently-captured controls selected from an adequately-large set of available samples.

### mcCNV and ExomeDepth perform comparably in a simulation study

To compare our mcCNV algorithm and ExomeDepth, we created synthetic pools of data across different sequencing depths. Based on our observations with the real data, we selected the total number of molecules for each sample from a uniform distribution defined as a 30% window on either side of the specified depth; for example, for a specified depth of 10 million molecules, we drew the molecules per sample from 7 to 13 million molecules. We used the observed capture probability at each exon from “Pool1” as the starting capture probability simplex for each simulation. For each depth ranging from 5 to 100 million molecules, we simulated 200 16-sample pools with single-exon variants. We allowed for homozygous and heterozygous deletions and duplications (0 to 4 copies), such that all variants were equally likely and the total variant probability was 1/1000. We used, as the starting capture probabilities (${\mathbb {E}}$), the empiric capture probabilities observed by summing across the Pool1 pool.

**Fig. 4:**
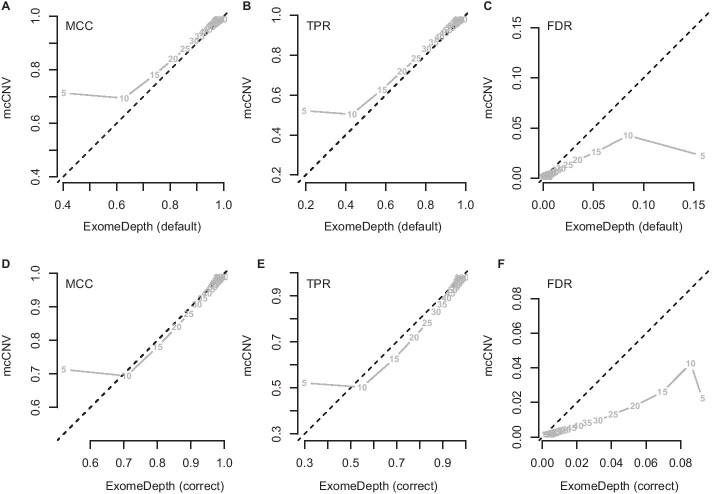
Algorithm performance comparing mcCNV and ExomeDepth. **a**–**c** mcCNV versus ExomeDepth with default parameters, 1/10, 000 transition probability and 50 kb expected variant length. **d**–**f** mcCNV versus ExomeDepth with simulation-matched parameters, 1/1000 transition probability and 1 kb expected variant length. Numbered points indicate the simulated depth in millions of molecules. ‘MCC’ indicates Matthew’s correlation coefficient; ‘TPR’ indicates true positive rate/sensitivity; ‘FDR’ indicates false discovery rate. Dashed black line shows the 1:1 relationship

We analyzed each of the 4000 pools (200 replicates by 20 depths) using our algorithm and two iterations of ExomeDepth. For the first iteration of ExomeDepth, we used the default values for transition probability (1/10, 000) and expected variant length (50 kb). For the second iteration, we used the true simulated variant prior for the transition probability (1/1000) and an expected variant length of 1 kb. As expected, the sensitivity increased, and the false discovery rate decreased as the sequencing depth increased (Fig. 4). In both comparisons, mcCNV demonstrated a lower false-discovery rate. When interrogating Matthew’s correlation coefficient [19] and the sensitivity, we found mcCNV had marginal performance over ExomeDepth with default parameters and marginal performance under ExomeDepth with simulation-matched parameters (table of values provided in supplemental materials).

**Fig. 5:**
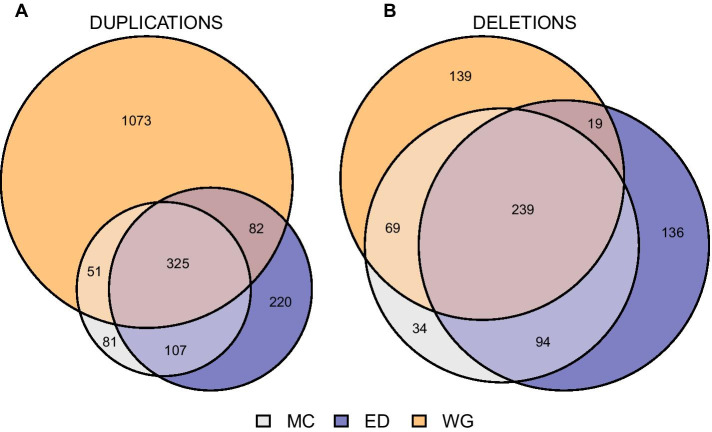
Copy number variant call concordance for the WGS pool, excluding subjects NCG_00790 and NCG_00851 due to poor correlation to the rest of the pool. **a** predicted duplications; **b** predicted deletions. mcCNV (MC) in grey; ExomeDepth (ED) in blue; ERDS/cnvpytor (WG) in orange. Values within overlaps give the number of variants

### mcCNV and ExomeDepth perform comparably on WGS pool

To compare mcCNV and ExomeDepth using real data, we performed matched genome sequencing on the subjects included in the WGS pool. Following the best practices suggested by Trost et al. [20], we performed read-depth-based CNV calling using the genome data. In line with recommendations by Trost et al., we excluded from comparative analysis any exons overlapping repetitive or low-complexity regions (34,856 out of 179,250). We then compared the exome calls using mcCNV and ExomeDepth to the genome calls using the overlap of ERDS [16] and cnvpytor [15]. Table 2 lists the total calls by subject. Overall, mcCNV predicted the largest number of variants; however, 85.7% of predicted variants were deletions from two samples (NCG_00790 and NCG_00851). ExomeDepth also predicted a disproportionate number of deletions for NCG_00790 and NCG_00851, totaling 69.4% of calls.

ExomeDepth only selected two and three controls for pools NCG_00790 and NCG_00851, respectively. Furthermore, NCG_00790 and NCG_00851 had substantially higher dispersion than the rest of the pool (two outliers in Fig. 3b).

Recognizing the genome calls do not represent an accurate truth set, we looked at mcCNV and ExomeDepth’s ability to predict the genome calls. Due to the large number of deletions called for NCG_00790 and NCG_00851, both algorithms performed poorly in predicting the genome calls (Table 3). When we excluded NCG_00790 and NCG_00851 from the analysis, mcCNV had comparable, uniformly better performance. Both algorithms demonstrated greater power to detect deletions. Figure 5 shows the call overlap, excluding NCG_00790 and NCG_00851, between the three approaches. Again excluding the two samples, we looked at the single-exon calls; 37.4% of mcCNV single-exon calls and 34.1% of ExomeDepth single-exon calls overlapped with the genome calls. We provide the full comparison by variant size in supplemental materials.

**Table 3:** mcCNV (MC)/ExomeDepth (ED) calls for ‘WGS’ pool (used as prediction) versus the ERDS/cnvpytor calls from matched genome sequencing (used as truth)

			MCC	TPR	FDR	PPV	BalAcc
DUP + DEL	Full	MC	0.18	0.34	0.90	0.10	0.67
		ED	0.26	0.36	0.81	0.19	0.68
	Sub	MC	0.49	0.34	0.31	0.69	0.67
		ED	0.48	0.38	0.38	0.62	0.69
DUP	Full	MC	0.40	0.24	0.33	0.67	0.62
		ED	0.35	0.24	0.50	0.50	0.62
	Sub	MC	0.40	0.25	0.33	0.67	0.62
		ED	0.38	0.27	0.45	0.55	0.63
DEL	Full	MC	0.18	0.64	0.95	0.05	0.82
		ED	0.22	0.56	0.91	0.09	0.78
	Sub	MC	0.68	0.66	0.29	0.71	0.83
		ED	0.54	0.55	0.47	0.53	0.78

Calls are subdivided by duplications (DUP) and deletions (DEL). ‘Full’ gives performance across the full pool; ‘Sub’ gives the performance excluding the poorly correlated samples NCG_00790 and NCG_00851 (gray rows). ‘MCC’ is Matthew’s correlation coefficient, ‘TPR’ is true positive rate/sensitivity, ‘FDR’ is false discovery rate, ‘PPV’ is positive predictive value, ‘BalAcc’ is balanced accuracy. Exons with any overlap of the repetitive and low-complexity regions, as defined in the Trost et al. manuscript, omitted from analysis

## Discussion

The medical genetics community still lacks robust exome-wide information about the prevalence of small (exon-level) variants. Others have established the reliability and cost-efficiency of pre-capture multiplexing [17, 21–24], and most commercial exome capture platforms have protocols for pre-capture multiplexing. Here, we demonstrate the reduction in inter-sample variance with pre-capture multiplexing, leading to increased power to detect exon-level copy number variation. Despite the benefits, many clinical laboratories do not employ a multiplexed capture protocol because multiplexing reduces capture efficiency [20] and requires waiting to fill a pool and may delay results. While we understand the increased complexity, multiplexed capture may uncover otherwise missed copy number variation and increase patients’ diagnostic yield.

Multiplexed capture is not without limitations. We presented an example (pool IDT-MC) where multiplexed capture provided little to no improvement over independently-captured samples. We concluded the absent improvement in inter-sample variance stemmed from the poor library balance before capture. Rebuilding a more-balanced pool with the same samples (pool IDT-RR) demonstrated a large reduction in inter-sample variance. Our example thus shows the importance of careful design when employing multiplexed capture.

In assessing the inter-sample variance, we compared two capture platforms: (1) Agilent SureSelectXT2 and (2) Integrated DNA Technologies xGen Lockdown Probes. We do not have enough data to suggest definitively one over the other. Comparing the mean-variance relationship, the IDT-RR pool appeared to have less dispersion overall (supplemental materials); however, the sample-specific dispersion estimates from ExomeDepth suggest better performance by the WGS pool (Fig. 3b). The higher pool-wide dispersion in the WGS pool comes from the two poorly correlated samples.

Our results suggest having a sufficiently large database of samples most-often provides appropriate control samples to estimate copy number variation (Fig. 3). However, we show laboratories can circumvent the need for large samples by multiplexing the capture step. Defining the capture pool as the set of controls both limits the need for regular reanalysis as the database grows and eliminates potential over-selecting of samples with the same variants.

With the read depths obtained for the WGS pool, our simplistic simulation study would suggest both mcCNV and ExomeDepth have the power to detect single-exon variants with >85% sensitivity while maintaining a low false-discovery rate (Fig. 4, supplemental materials). However, comparing the exome calls to the genome calls for the WGS pool revealed lackluster concordance. As Trost et al. point out, the genome CNV callers still struggle with variants less than 1 kb [20]. Considering the poor performance of genome-based callers on small variants and the exome collection parameters, the exome results may provide greater reliability than the genome results. However, given the distribution of calls throughout the exome, we dismiss the thousands of excess deletions called for NCG_00790 and NCG_00851. The excess deletions observed likely stem from DNA degradation, but we lack additional DNA to confirm suspected input quality issues. Confirmation of the individual calls is beyond the scope of this work.

Unsurprisingly, both mcCNV and ExomeDepth failed to call many of the duplications called from the genome data. The variance for the negative binomial increases as the mean increases; we expect greater variation in read depth from duplicated loci, making duplications more difficult to distinguish. Similarly, the variance of the binomial proportion increases monotonically over [0, 0.5). More sensitive detection of duplications will likely require greater sequencing depth.

With comparable performance, we emphasize two strengths of using the mcCNV algorithm. First, the algorithm does not require any user-defined prior information, whereas ExomeDepth requires prior information about both the prevalence and the size of copy number variants. Second, the analysis occurs solely at the exon level. While the mcCNV approach does not define the variant breakpoints, the resulting model does not include bias from fragment length/GC correction.

The simulation study emphasizes the importance of sequencing depth (in terms of absolute molecules). We can collect increased basepair coverage for less money by sequencing longer reads (e.g. 2 × 150 vs. 2 × 50), but doing so decreases power for depth-based CNV calling. The sequencing depth in clinical exomes varies widely between efforts, with average depths in the Clinical Sequencing Exploratory Research (CSER) consortium ranging from 63-233x [25]; others have suggested an ideal depth of 120x for SNP/indel calling [26]. We demonstrate the need for deeper sequencing if we wish to establish exon-level variants.

Additionally, we recognize the increased capture efficiency in hard-to-capture regions using independent captures; multiplexing the capture step reduces the capture efficiency by 20-30% [20]. We feel the variance benefit of multiplexed capture outweighs the decrease in capture efficiency. Without an accurate estimate of the disease burden caused by exon-level CNVs, we cannot comment on the cost-benefit of multiplexed capture with adequate sequencing depth. Until greater information exists, we advocate for multiplexed capture and deep sequencing to identify small CNVs.

We believe the uncertainty about the prevalence and clinical significance of exon-level variants warrants a large undertaking. Even if we take the conservative approach and look only at concordant calls between genome and exome sequencing (Fig. 5), we have an average of 40 variants per sample to contend with. Two possibilities exist: (1) the algorithms all fail over specific regions, or (2) some genes can tolerate intragenic copy-number variation better than others. Having eliminated calls from repetitive and low-complexity regions, we believe possibility (2) is more likely. To truly determine the prevalence (and therefore, clinical significance) of exon-level variants we need to interrogate exon-level variants on a large cohort. Confirmation testing for the tens to thousands of predicted variants from the exome and genome calls would allow true determination of algorithm performance and inform the clinical utility.

## Conclusions

Taken together, we recommend the following: (1) research and clinical endeavors consider adjusting protocols to multiplex samples before any targeted capture; (2) before capture, we suggest checking the library balance and adjusting as necessary (we achieved good performance when the relative standard deviation of sequenced molecules per sample fell below 25%); (3) collecting an average of 225 filtered read-pairs per target. We then provide a simple-to-use and efficient R package to estimate copy number utilizing the negative binomial distribution.

## Methods

### Exome sequencing

We performed sequencing on human samples of purified DNA obtained from the Wilhelmsen laboratory collection, the NCGENES cohort [27], and the Coriell Institute in compliance with all guidelines and regulations under the supervision of the UNC Institutional Review Board. We also utilized existing read-level data from the NCGENES [27] project. All human data were collected following all guidelines and regulations with the approval and under the supervision of the UNC Institutional Review Board. All research participants, or participants’ guardians when applicable, received appropriate counseling and provided informed consent to participate in this research. No identifying information or sequence level data are included in this manuscript or accompanying data.

We compared the performance of two capture platforms: (1) Agilent SureSelect XT2 (multiplexed capture)/Agilent SureSelect XT (independent capture); (2) Integrated DNA Technologies (IDT) xGen Lockdown Probes. We utilized Human All Exome v4 baits (Agilent) and Exome Research Panel v1 baits (IDT). All captures performed according to manufacturer protocol, with the following exceptions: (1) we multiplexed 16 samples versus the recommended 8 for the XT2 protocol for some pools; (2) for Pool2, we performed the fragmentation step 5 times to test whether a more uniform fragment length distribution would improve capture.

All sequencing performed with Illumina (2 × 100) paired-end chemistry with one exception: we initially sequenced the “WGS” pool with 2 × 150 chemistry then collected additional sequencing on the same library using 2 × 50 chemistry. We aligned paired reads to hg19v0 (GATK resource bundle) using BWA-MEM [28] and removed duplicate reads using Picard tools. We then used our novel R package, mcCNV, to count the number of overlapping molecules (read-pairs) per exon. For inclusion, we required properly-paired molecules with unambiguous mapping for one read and mapping quality greater than or equal to 20 for both reads. Full Snakemake [29] pipeline provided in supplemental materials. Table 1 provides an overview of the exome sequencing included.

The pool names can be considered arbitrary. Briefly, “Pool1/2” were the first pools we sequenced, “SMA1/2” include samples with known deletions in the SMN1 gene (not covered by either capture platform used), “IDT-MC/IDT-IC” indicate multiplexed and independent capture pools using the IDT platform, “IDT-RR” is the re-capture and re-sequencing of the “IDT-MC” samples, and “WGS” is the pool with matched whole-genome sequencing.

### Genome sequencing

For the 16 samples in the “WGS” pool, we performed genome sequencing using Illumina 2 × 150 chemistry to an average 50 × coverage. The low available input DNA required PCR amplification during library preparation. We followed Trost et al. recommendations for making read-depth based CNV calls [20]. Briefly, we mapped paired-reads identical to our targeted sequencing data. We then interrogated the read depth interquartile range using samtools depth [30], recalibrated base-quality scores and called sequence variants using GATK [31], and called copy number variants using the ERDS [16] and cnvpytor (updated implementation of CNVnator) [15] algorithms. Full Snakemake [29] pipeline provided in supplemental materials.

### Simulating targeted sequencing

A multinomial process models repeated independent trials with distinct outcomes, each outcome having a set probability (e.g., rolling a die ten times). To simulate the capture in targeted sequencing, we model each molecule captured as a multinomial trial with a possible outcome for each targeted region. To define the subject-specific multinomial distribution, we start with a shared probability simplex giving the baseline capture probability at each target. We then multiply the baseline probability by the subject-specific copy state at each target and normalize, giving the subject-specific multinomial distribution. We use an alternate definition of copy state, such that 1 represents the normal diploid state.

Formally, let $e_j \in {\mathbb {E}}$ represent the baseline probability of capturing target *j* and $n_i$ represent the total number of molecules (read pairs) for subject *i*. For each subject, *i*:

1. Randomly select $s_{ij} \in {\mathbb {S}}_i$ from $S = \{0.0, 0.5, 1, 1.5, 2\}$ as the copy number at target *j*
2. Adjust the subject-specific capture probabilities by the copy number, ${\mathbb {E}}_i = \frac{{\mathbb {E}} \odot {\mathbb {S}}_{i}}{\sum _j {\mathbb {E}} \odot {\mathbb {S}}_{i}}$
3. Draw $n_i$ times from $\text {Multinomial}({\mathbb {E}}_i)$, giving the molecule counts at each target *j* for sample *i*, $c_{ij} \in {\mathbb {C}}_i$

We provide functionality within the mcCNV R package for producing reproducible simulations. Note, the user must provide ${\mathbb {E}}$ (the baseline/un-adjusted probability of capture). The mcCNV R package includes functionality for randomly defining ${\mathbb {E}}$, but the simulations included in this work used the observed capture probabilities from “Pool1.”

### mcCNV algorithm

The mcCNV algorithm was adapted from the sSEQ method for quantifying differential expression in RNA-seq experiments with small sample sizes [32]. Yu et al. provide detailed theoretical background of the negative binomial model and using shrinkage to improve dispersion estimates. The mcCNV algorithm adjusts the sSEQ probability model by adding a multiplier for the copy state:

1 $$\begin{aligned} C_{ij} \sim {\mathcal {N}}{\mathcal {B}}(f_is_{ij}{{\hat{\mu }}}_j, {\tilde{\phi }}_j/f_i) \end{aligned}$$

where the random variable $C_{ij}$ represents observed molecule counts for subject *i* at target *j*, $f_i$ is the size factor for subject *i*, $s_{ij}$ is the copy state, $\mu _j$ is the expected mean under the diploid state at target *j*, and ${\tilde{\phi }}_j$ is the shrunken phi at target *j*. We observe $c_{ij}$ and wish to estimate $s_{ij}$, ${\hat{s}}_{ij}$. Initialize by setting ${\hat{s}}_{ij} = 1$ for all *i*, *j*. Then,

1. Adjust the observed values for the estimated copy-state,
  2 $$\begin{aligned} c_{ij}^{\prime } = \frac{c_{ij}}{{\hat{s}}_{ij}}. \end{aligned}$$
2. Subset $c_{ij}^{\prime }$ such that $c_{ij}^{\prime }> 10, ~ {\hat{s}}_{ij} > 0$
3. Calculate the size-factor for each subject
  3 $$\begin{aligned} f_i = \text {median}\left( \frac{c_{ij}^{\prime }}{g_j}\right) , \end{aligned}$$
  where $g_j$ is the geometric mean at target *j*.
4. Use method of moments to calculate the expected dispersion
  4 $$\begin{aligned} {\hat{\phi }}_j = \max \left( 0, \frac{{\hat{\sigma }}_j^2 - \hat{\mu }_j}{\hat{\mu }_j^2}\right) \end{aligned}$$
  where $\hat{\mu }_j$ and $\hat{\sigma }_j^2$ are the sample mean and variance of $c_{ij}^{\prime }/f_i$.
5. Let *J* represent the number of targets. Shrink the phi values to
  5 $$\begin{aligned} {\tilde{\phi }}_j = (1 - \delta ){\hat{\phi }}_j + \delta \hat{\xi } \end{aligned}$$
  such that
  6 $$\begin{aligned} \delta = \frac{\sum \limits _j\left( {\hat{\phi }}_j - \frac{1}{n_j}\sum \limits _j {\hat{\phi }}_j\right) ^2/(J - 1)}{\sum \limits _j\left( {\hat{\phi }}_j - \hat{\xi }\right) ^2/(n_j - 2)} \end{aligned}$$
  and
  7 $$\begin{aligned} \hat{\xi } = \mathop {\text {argmin}}\limits _{\xi }\left\{ \frac{d}{d\xi }\frac{1}{\sum \limits _j \left( {\hat{\phi }}_j - \xi \right) ^2} \right\} . \end{aligned}$$
6. Update $${\hat{s}}_{ij}$$,
  8 $$\begin{aligned} \mathop {\text {argmax}}\limits _{s \in S}\left\{ \mathcal {L}(s |c_{ij},f_i,{\hat{\mu }}_j,{\tilde{\phi }}_j) \right\} \end{aligned}$$
  where $$S = \{0.001, 0.5, 1, 1.5, 2\}$$.
7. Repeat until the number of changed states falls below a threshold or a maximum number of iterations is reached.
8. After convergence, calculate *p* values for the diploid state, $\pi _{ij} = \text {Pr}(s_{ij} = 1)$.
9. Adjust *p* values using the Benjamini-Hochberg procedure [33] and filter to a final call-set such that adjusted *p* values fall below some threshold, $\alpha$.

## Supplementary Information

**Additional file 1.** Vignette showing the R code and scripts necessary to reproduce the analysis presented.

## Data Availability

mcCNV is implemented as an R package: https://github.com/daynefiler/mcCNV. All data and functionality to produce this manuscript provided in a standalone R package with a vignette replicating the analysis: https://github.com/daynefiler/filer2020A.
