# Supplementary material for: Pre-capture multiplexing provides additional power to detect copy number variation in exome sequencing
Source: BMC Bioinformatics. 2021 Jul 20;22:374. doi: 10.1186/s12859-021-04246-w (PMC8293537; doi:10.1186/s12859-021-04246-w)
Supplement: Supplementary file 1 — Additional file 1. Vignette showing the R code and scripts necessary to reproduce the analysis presented. [file 12859_2021_4246_MOESM1_ESM.pdf]

Pre-capture multiplexing provides additional power to detect copy number variation in exome sequencing: Supplemental Information

Dayne L. Filer[1,2], Fengshen Kuo[1], Alicia T. Brandt[1], Christian R. Tilley[1], Piotr A. Mieczkowski[1], Jonathan S. Berg[1], Kimberly Robasky[1,2,3], Yun Li[1,4], Chris Bizon[2], Jeffery L. Tilson[2], Bradford C. Powell[1,2], Darius M. Bost[1,2], Clark D. Jeffries[2], and Kirk C. Wilhelmsen[1,2,5]

[1]Department of Genetics, UNC School of Medicine, Chapel Hill, NC
[2]Renaissance Computing Institute, Chapel Hill, NC
[3]UNC School of Information and Library Science, Chapel Hill, NC
[4]Department of Biostatistics, UNC Gillings School of Global Public Health, Chapel Hill, NC
[5]Department of Neurology, UNC School of Medicine, Chapel Hill, NC

Contents

1 Setup **2**

2 Summary of data **2**

3 Mean-variance relationship for multplexed versus independent capture **3**

4 ExomeDepth selection **7**

5 Comparing calls on simulation study **8**

6 Comparing calls on WGS pool **10**

7 Slurm scripts to perform simulation study **15**

7.1 Create count objects . . . 15
7.2 Run mcCNV on count objects . . . 16
7.3 Run ExomeDepth with default parameters on count objects . . . 18
7.4 Run ExomeDepth with simulation-matched parameters on count objects . . . 19

8 Snakemake notes **20**

9 Snakemake file for exome anlaysis **22**

10 Snakemake file for genome anlaysis **24**

1 Setup

```
library(filer2020A)
library(mcCNV)
library(eulerr)
library(dlfUtils)
library(parallel)
library(grid)
```

2 Summary of data

```
data(subjectMeta)
poolTbl <- subjectMeta[ ,
                        .(N = .N,
                          medExon = round(median(medIntMolCount), 0),
                          medTotal = round(median(totalMolCount), 0),
                          minTotal = min(totalMolCount),
                          maxTotal = max(totalMolCount),
                          rsdTotal = sd(totalMolCount)/mean(totalMolCount)*100),
                        by = .(pool, capture, multiplexCapture)]
poolTbl[ , rsdTotal := round(rsdTotal, 1)]
poolTbl

##       pool capture multiplexCapture   N medExon medTotal minTotal  maxTotal
## 1:  IDT-IC     IDT            FALSE  16     143 55149058 37453015  85138915
## 2:  IDT-MC     IDT             TRUE  16      93 29772684 16674468 118147912
## 3:  IDT-RR     IDT             TRUE  16     272 79079629 61289322 120147888
## 4: NCGENES Agilent            FALSE 112      93 24451245 12749793  68565471
## 5:   Pool1 Agilent             TRUE  16      56 13265614  8911132  17324903
## 6:   Pool2 Agilent             TRUE  16      86 21076056  4585195  27846146
## 7:    SMA1 Agilent             TRUE   8      56 12256002 11051840  13600697
## 8:    SMA2 Agilent             TRUE   8      25  5622040  4904000   6545360
## 9:     WGS Agilent             TRUE  16     196 46406224 36496097  65200410
##    rsdTotal
## 1:     22.4
## 2:     64.2
## 3:     22.9
## 4:     27.6
## 5:     18.5
## 6:     27.6
## 7:      6.2
## 8:     10.4
## 9:     16.4
```

3 Mean-variance relationship for multplexed versus independent capture

To show the difference in independent and multiplexed captures, we randomly select pools of samples independently-captured using the same platform. We then calculate interval statistics (mean, variance, etc.) across the pools.

```
smplSubject <- function(poolName, n) {
  data(subjectMeta, envir = environment())
  subjectMeta[pool == poolName, sample(subject, n, replace = FALSE)]
}
set.seed(1234)
pools <- c(replicate(5, smplSubject("NCGENES", 16), simplify = FALSE))
names(pools) <- c(sprintf("randNCG_%d", 1:5))
pools <- c(pools,
           with(subjectMeta[pool != "NCGENES"], split(subject, pool)))
```

```
## Calculate mean-variance by pool
mnvr <- mclapply(pools, subsetCounts, mc.cores = length(pools))
mnvr <- mclapply(mnvr, calcIntStats, mc.cores = length(mnvr))
for (i in seq_along(mnvr)) {
  mnvr[[i]][ , pool := names(mnvr)[i]]
}
mnvr <- rbindlist(mnvr)
setkey(mnvr, pool); setcolorder(mnvr)

## Estimate alpha0
alpha0 <- mclapply(pools, estAlpha0, mc.cores = length(pools))
```

```
a0tbl <- data.table(pool = names(alpha0),
                    a0 = sapply(alpha0, "[[", "a0"),
                    N = sapply(alpha0, "[[", "N"))
a0tbl[ , aMn := a0/N]
calcRange <- function(x) {
  subjectMeta[subject %in% x,
              .(mnCount = min(totalMolCount),
                mdCount = median(totalMolCount),
                mxCount = max(totalMolCount),
                rsCount = sd(totalMolCount)/mean(totalMolCount)*100)]
}
poolCts <- lapply(pools, calcRange)
poolCts <- lapply(names(poolCts), function(x) poolCts[[x]][ , pool := x])
poolCts <- rbindlist(poolCts)
a0tbl <- merge(a0tbl, poolCts)
a0tbl[ , mc := !grepl("IDT-IC|rand", pool)]
a0tbl[ , idt := grepl("IDT", pool)]
```

```r
pltAlpha0(a0tbl)
```

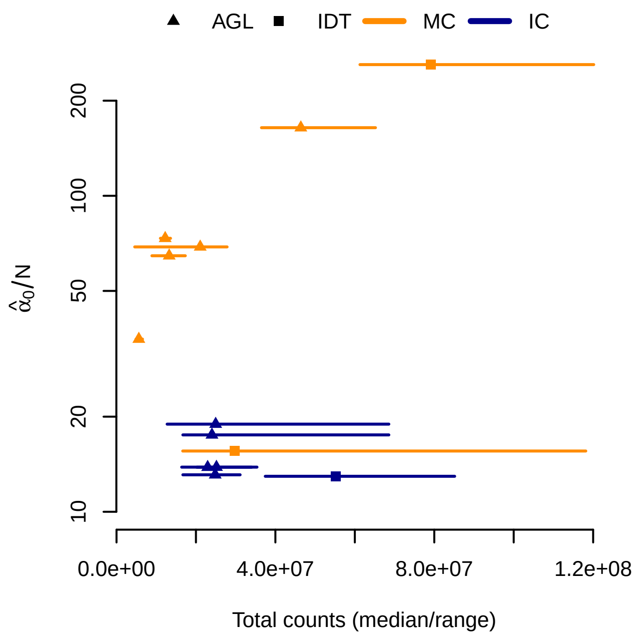

Figure S1: manuscript figure

```r
aglPools <- c(sprintf("randNCG_%d", 1:5), "Pool1", "Pool2", "WGS", "SMA1", "SMA2")
with(mnvr[pool %in% aglPools], {
  pltMnVrCont(dat = as.data.table(as.list(environment())),
              grpVec = factor(pool, levels = aglPools),
              colVec = c(rep('darkblue', 5), rep('darkorange', 5)),
              lgnd = FALSE)
})
addfiglab("A", units = "ndc")
legend(x = "center", lwd = 4, col = c('darkblue', 'darkorange'),
       c("AGL-IC", "AGL-MC"), bty = "n", cex = 0.75)
idtPools <- c("IDT-MC", "IDT-IC", "IDT-RR")
with(mnvr[pool %in% idtPools], {
  pltMnVrCont(dat = as.data.table(as.list(environment())),
              grpVec = factor(pool, levels = idtPools),
              colVec = c("darkorange", "darkblue", "darkorange"),
              lgnd = FALSE)
})
addfiglab("B")
legend(x = "center", lwd = 4, col = c('darkblue', 'darkorange'),
       c("IDT-IC", "IDT-MC"), bty = "n", cex = 0.75)
with(mnvr[pool %in% c("WGS", "IDT-RR")], {
  pltMnVrCont(dat = as.data.table(as.list(environment())),
              grpVec = factor(pool, levels = c("WGS", "IDT-RR")),
              colVec = c("darkblue", "darkorange"))
})
```

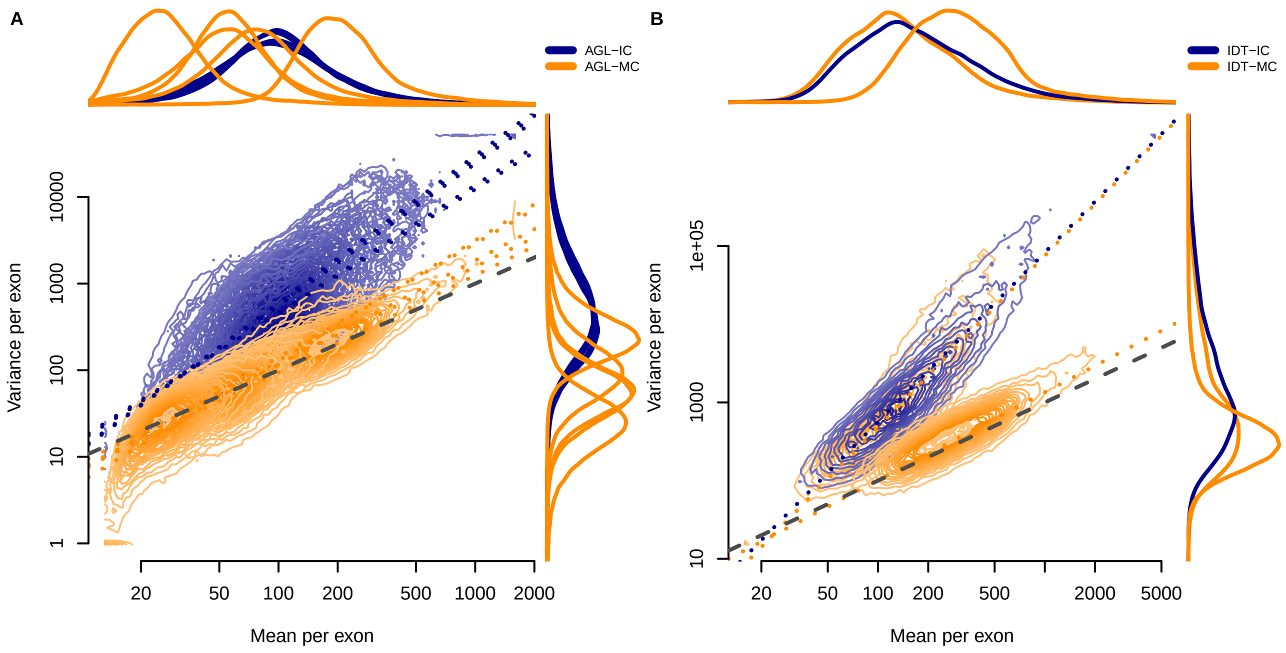

Figure S2: manuscript figure

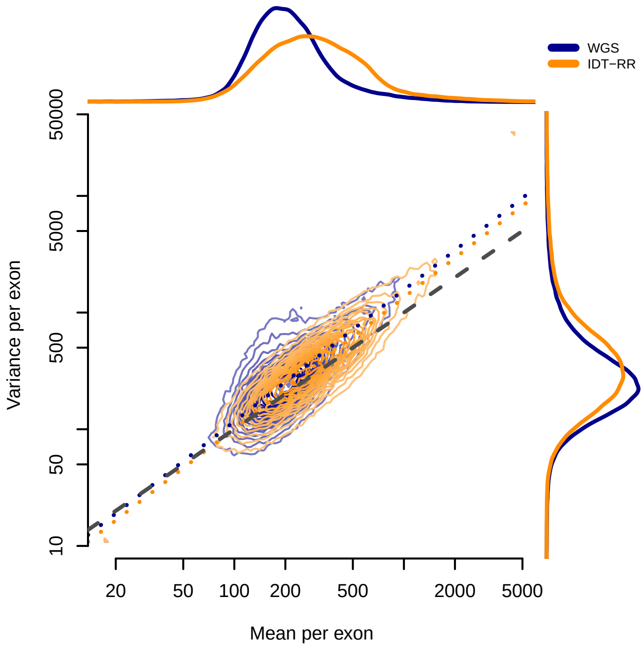

Figure S3: Comparison of mean-variance relationship between WGS pool (blue) and IDT-RR pool (orange). Mean count by exon given on horizontal axis; variance of exon counts given on horizontal axis. Dotted lines show the ordinary least-squares fit. Lines above plot show the distribution of mean values; lines to the right of the plot show the distribution of variance values.

4 ExomeDepth selection

```
pltSubjectStatByPool("medIntMolCount", ylab = "Median count per exon")
addfiglab("A")
pltSubjectStatByPool("overallPhi", ylab = "Overdispersion (phi)")
addfiglab("B")
pltSubjectStatByPool("propSelected", ylab = "Proportion of controls selected")
addfiglab("C")
pltSubjectStatByPool("nSelected", ylab = "Number of controls selected")
addfiglab("D")
```

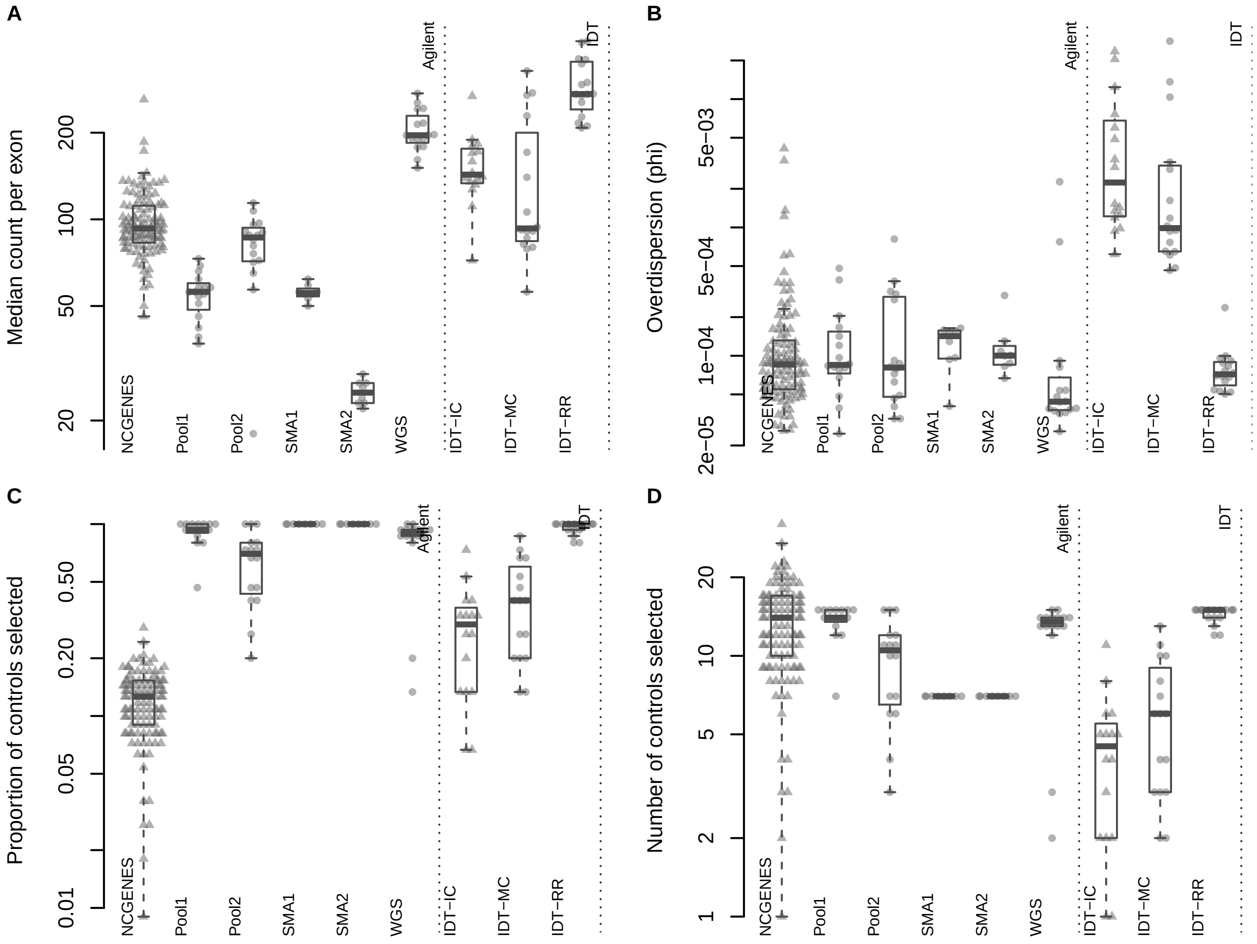

Figure S4: manuscript figure

5 Comparing calls on simulation study

```
data(simRes)
procSimRes <- lapply(simRes, function(x) procRes(x$clpRes))

simResTbl <- lapply(procSimRes,
                    function(x) x$mnDat[ , .(mcc, tpr, fdr), keyby = dep])
simResTbl <- Reduce(merge, simResTbl)
setnames(simResTbl,
         c("dep",
           "mcMCC", "mcTPR", "mcFDR",  ## mcCNV
           "edMCC", "edTPR", "edFDR",  ## ExomeDepthDefault
           "ebMCC", "ebTPR", "ebFDR")) ## ExomeDepthBest
setcolorder(simResTbl,
            c("dep", "mcMCC", "edMCC", "ebMCC", "mcTPR",
              "edTPR", "ebTPR", "mcFDR", "edFDR", "ebFDR"))
simResTbl <- simResTbl[ , lapply(.SD, signif, 3), by = dep]
simResTbl

##     dep mcMCC edMCC ebMCC mcTPR edTPR ebTPR   mcFDR   edFDR   ebFDR
##  1:   5 0.713 0.401 0.519 0.522 0.192 0.298 0.02230 0.15900 0.09260
##  2:  10 0.694 0.628 0.708 0.503 0.431 0.549 0.04250 0.08450 0.08590
##  3:  15 0.781 0.742 0.801 0.627 0.581 0.690 0.02600 0.05270 0.06940
##  4:  20 0.840 0.811 0.857 0.719 0.682 0.777 0.01810 0.03420 0.05360
##  5:  25 0.879 0.856 0.893 0.783 0.752 0.832 0.01310 0.02460 0.04090
##  6:  30 0.907 0.889 0.918 0.831 0.804 0.872 0.00967 0.01750 0.03210
##  7:  35 0.926 0.909 0.935 0.864 0.839 0.897 0.00807 0.01370 0.02600
##  8:  40 0.941 0.927 0.948 0.891 0.869 0.917 0.00638 0.01060 0.02080
##  9:  45 0.952 0.940 0.957 0.911 0.892 0.932 0.00527 0.00846 0.01680
## 10:  50 0.961 0.950 0.965 0.927 0.910 0.944 0.00437 0.00701 0.01370
## 11:  55 0.966 0.957 0.969 0.937 0.921 0.951 0.00377 0.00569 0.01180
## 12:  60 0.972 0.963 0.974 0.947 0.933 0.959 0.00318 0.00517 0.00986
## 13:  65 0.976 0.969 0.978 0.955 0.943 0.964 0.00290 0.00433 0.00837
## 14:  70 0.978 0.972 0.980 0.960 0.949 0.968 0.00252 0.00381 0.00735
## 15:  75 0.981 0.976 0.983 0.965 0.955 0.972 0.00212 0.00321 0.00625
## 16:  80 0.983 0.978 0.985 0.969 0.960 0.975 0.00200 0.00294 0.00560
## 17:  85 0.985 0.980 0.986 0.972 0.963 0.977 0.00181 0.00263 0.00491
## 18:  90 0.987 0.982 0.987 0.975 0.967 0.979 0.00169 0.00243 0.00451
## 19:  95 0.988 0.984 0.988 0.978 0.970 0.981 0.00156 0.00223 0.00393
## 20: 100 0.989 0.985 0.989 0.980 0.973 0.982 0.00150 0.00195 0.00359
```

```
pltStatCompare(xRes = procSimRes$ExomeDepthDefault, yRes = procSimRes$mcCNV,
               stat = "mcc", xlab = "ExomeDepth (default)", ylab = "mcCNV")
addfiglab("A")
pltStatCompare(xRes = procSimRes$ExomeDepthDefault, yRes = procSimRes$mcCNV,
               stat = "tpr", xlab = "ExomeDepth (default)", ylab = "mcCNV")
addfiglab("B")
pltStatCompare(xRes = procSimRes$ExomeDepthDefault, yRes = procSimRes$mcCNV,
               stat = "fdr", xlab = "ExomeDepth (default)", ylab = "mcCNV")
addfiglab("C")
```

```
pltStatCompare(xRes = procSimRes$ExomeDepthBest, yRes = procSimRes$mcCNV,
               stat = "mcc", xlab = "ExomeDepth (correct)", ylab = "mcCNV")
addfiglab("D")
pltStatCompare(xRes = procSimRes$ExomeDepthBest, yRes = procSimRes$mcCNV,
               stat = "tpr", xlab = "ExomeDepth (correct)", ylab = "mcCNV")
addfiglab("E")
pltStatCompare(xRes = procSimRes$ExomeDepthBest, yRes = procSimRes$mcCNV,
               stat = "fdr", xlab = "ExomeDepth (correct)", ylab = "mcCNV")
addfiglab("F")
```

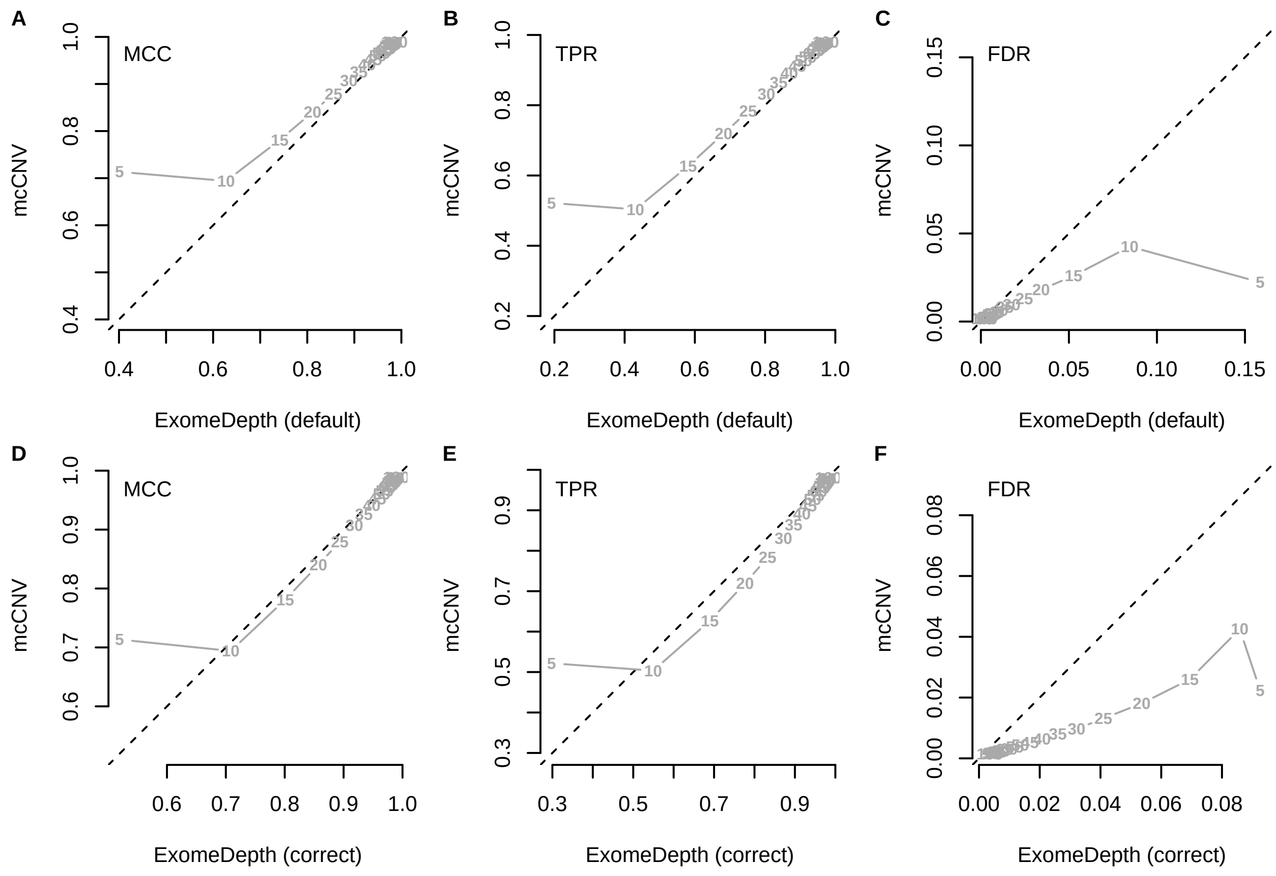

Figure S5

6 Comparing calls on WGS pool

```
data(wgsPoolCalls)
mergeAll <- function(x, y) merge(x, y, all = TRUE)
wgs <- Reduce(mergeAll, wgsPoolCalls)
data(intAgl)
xpandInt <- function(int, sbjVec) {
  lst <- vector(mode = "list", length = length(sbjVec))
  names(lst) <- sbjVec
  for (s in sbjVec) {
    lst[[s]] <- copy(int)
    lst[[s]][ , subject := s]
  }
  rbindlist(lst)
}
wgsAgl <- xpandInt(intAgl, wgs[ , unique(subject)])
setkeyv(wgsAgl, key(wgs))
wgs <- wgs[wgsAgl]
rm(wgsAgl)
wgs <- wgs[!(rlcr),
           .(mcDup = !is.na(passFilter) & CN > 1,
             edDup = !is.na(type) & type == "duplication",
             wgDup = !is.na(erds) & !is.na(cnvpytor) & erds == "dup",
             mcDel = !is.na(passFilter) & CN < 1,
             edDel = !is.na(type) & type == "deletion",
             wgDel = !is.na(erds) & !is.na(cnvpytor) & erds == "del"),
           by = .(subject, seqnames, start, end)]
wgs[ , mc := mcDup | mcDel]
wgs[ , ed := edDup | edDel]
wgs[ , wg := wgDup | wgDel]
setcolorder(wgs, c(key(wgs), 'mc', 'ed', 'wg'))
wgsCallBySbj <- wgs[ , lapply(.SD, sum), .SDcols = is.logical, by = subject]
wgsCallBySbj

##       subject   mc   ed  wg mcDup edDup wgDup mcDel edDel wgDel
##  1: NCG_00012   90  106 143    61    73   121    29    33    22
##  2: NCG_00237   82  101 165    50    64   129    32    37    36
##  3: NCG_00525   68   74 151    30    33   110    38    41    41
##  4: NCG_00593   45   58 142    22    28    81    23    30    61
##  5: NCG_00676   66   78 112    38    46    92    28    32    20
##  6: NCG_00790 5156 2204 121    19    37    92  5137  2167    29
##  7: NCG_00819   68   76 134    30    41   100    38    35    34
##  8: NCG_00840   78   92 157    44    52   115    34    40    42
##  9: NCG_00851 1151  859 141    28    51   102  1123   808    39
## 10: NCG_00857   59   75 119    10    15    81    49    60    38
## 11: NCG_00976   46   58 114    25    37    93    21    21    21
## 12: NCG_01023   59   95 143    32    60   113    27    35    30
## 13: NCG_01043   73   94 128    40    64   105    33    30    23
## 14: NCG_01076   36   57 105     7    22    78    29    35    27
## 15: NCG_01077  135  157 230   103   121   184    32    36    46
## 16: NCG_01117   95  101 154    72    78   129    23    23    25
```

```
wgs[ ,
    c("mcRunID", "edRunID", "wgRunID") :=
      .(paste(subject, seqnames, rleid(mc), sep = ":"),
        paste(subject, seqnames, rleid(ed), sep = ":"),
        paste(subject, seqnames, rleid(wg), sep = ":")),
    by = .(subject, seqnames)]
wgs[!(mc), mcRunID := NA_character_]
wgs[!(ed), edRunID := NA_character_]
wgs[!(wg), wgRunID := NA_character_]
wgs[(mc), mcVarLen := .N, by = .(mcRunID)]
wgs[(ed), edVarLen := .N, by = .(edRunID)]
wgs[(wg), wgVarLen := .N, by = .(wgRunID)]

## Count number of variants by variant length for each algorithm
mergeAll <- function(x, y) merge(x, y, all = TRUE)
wgs[!subject %in% c("NCG_00790", "NCG_00851"), {
  lst <- list(.SD[!is.na(mcVarLen),
                  .(mc = length(unique(mcRunID))),
                  by = .(varLen = pmin(mcVarLen, 10))],
              .SD[!is.na(edVarLen),
                  .(ed = length(unique(edRunID))),
                  by = .(varLen = pmin(edVarLen, 10))],
              .SD[!is.na(wgVarLen),
                  .(wg = length(unique(wgRunID))),
                  by = .(varLen = pmin(wgVarLen, 10))])
  Reduce(mergeAll, lst)
}]

##     varLen  mc  ed  wg
##  1:      1 297 179 138
##  2:      2  78  78  78
##  3:      3  57  71 102
##  4:      4  14  17  36
##  5:      5  15  17  30
##  6:      6  12  20  15
##  7:      7   5   5   4
##  8:      8   4   6  25
##  9:      9   2   1  15
## 10:     10   6  13  42

calcOverlap <- function(vl, exclude = c("NCG_00790", "NCG_00851")) {
  mc <- wgs[!subject %in% exclude][pmin(mcVarLen, 10) == vl]
  mc <- mc[ , .(wg = any(wg), ed = any(ed)), by = .(mcRunID)]
  mc <- mc[ , .(vl = vl, prd = "mc", wg = sum(wg)/.N, ed = sum(ed)/.N)]
  mc <- melt(mc, id.vars = c("vl", "prd"))

  ed <- wgs[!subject %in% exclude][pmin(edVarLen, 10) == vl]
  ed <- ed[ , .(wg = any(wg), mc = any(mc)), by = .(edRunID)]
  ed <- ed[ , .(vl = vl, prd = "ed", wg = sum(wg)/.N, mc = sum(mc)/.N)]
  ed <- melt(ed, id.vars = c("vl", "prd"))
```

```
  wg <- wgs[!subject %in% exclude][pmin(wgVarLen, 10) == vl]
  wg <- wg[ , .(mc = any(mc), ed = any(ed)), by = .(wgRunID)]
  wg <- wg[ , .(vl = vl, prd = "wg", mc = sum(mc)/.N, ed = sum(ed)/.N)]
  wg <- melt(wg, id.vars = c("vl", "prd"))

  res <- rbindlist(list(mc, ed, wg))
  res[ , .(vl, prd, tst = as.character(variable), pct = round(value, 3))][]
}
## Percentage of calls predicted (prd) found in the other algorithms (tst)
## by variant length (vl)
callByVarLen <- rbindlist(lapply(1:10, calcOverlap))[order(vl, prd, tst)]
cbind(callByVarLen[1:30], callByVarLen[31:60])

##     vl prd tst   pct vl prd tst   pct
##  1:  1  ed  mc 0.732  6  ed  mc 1.000
##  2:  1  ed  wg 0.341  6  ed  wg 0.650
##  3:  1  mc  ed 0.700  6  mc  ed 1.000
##  4:  1  mc  wg 0.374  6  mc  wg 0.917
##  5:  1  wg  ed 0.391  6  wg  ed 0.800
##  6:  1  wg  mc 0.478  6  wg  mc 1.000
##  7:  2  ed  mc 0.808  7  ed  mc 0.800
##  8:  2  ed  wg 0.577  7  ed  wg 0.600
##  9:  2  mc  ed 0.885  7  mc  ed 1.000
## 10:  2  mc  wg 0.615  7  mc  wg 1.000
## 11:  2  wg  ed 0.654  7  wg  ed 0.250
## 12:  2  wg  mc 0.538  7  wg  mc 0.250
## 13:  3  ed  mc 0.831  8  ed  mc 1.000
## 14:  3  ed  wg 0.662  8  ed  wg 0.500
## 15:  3  mc  ed 0.912  8  mc  ed 1.000
## 16:  3  mc  wg 0.737  8  mc  wg 1.000
## 17:  3  wg  ed 0.392  8  wg  ed 0.240
## 18:  3  wg  mc 0.451  8  wg  mc 0.160
## 19:  4  ed  mc 1.000  9  ed  mc 1.000
## 20:  4  ed  wg 0.471  9  ed  wg 1.000
## 21:  4  mc  ed 1.000  9  mc  ed 1.000
## 22:  4  mc  wg 1.000  9  mc  wg 1.000
## 23:  4  wg  ed 0.278  9  wg  ed 0.067
## 24:  4  wg  mc 0.444  9  wg  mc 0.067
## 25:  5  ed  mc 0.882 10  ed  mc 1.000
## 26:  5  ed  wg 0.353 10  ed  wg 0.846
## 27:  5  mc  ed 1.000 10  mc  ed 1.000
## 28:  5  mc  wg 1.000 10  mc  wg 1.000
## 29:  5  wg  ed 0.433 10  wg  ed 0.310
## 30:  5  wg  mc 0.433 10  wg  mc 0.310
##     vl prd tst   pct vl prd tst   pct
```

```
pmLst <- list()
pmLst$mc <- with(wgs, evalPred(mc, wg))
pmLst$ed <- with(wgs, evalPred(ed, wg))
pmLst$mcSub <- with(wgs[!grepl("790|851", subject)], evalPred(mc, wg))
```

```r
pmLst$edSub <- with(wgs[!grepl("790|851", subject)], evalPred(ed, wg))
pmLst$mcDup <- with(wgs, evalPred(mcDup, wgDup))
pmLst$edDup <- with(wgs, evalPred(edDup, wgDup))
pmLst$mcSubDup <- with(wgs[!grepl("790|851", subject)],
                       evalPred(mcDup, wgDup))
pmLst$edSubDup <- with(wgs[!grepl("790|851", subject)],
                       evalPred(edDup, wgDup))
pmLst$mcDel <- with(wgs, evalPred(mcDel, wgDel))
pmLst$edDel <- with(wgs, evalPred(edDel, wgDel))
pmLst$mcSubDel <- with(wgs[!grepl("790|851", subject)],
                       evalPred(mcDel, wgDel))
pmLst$edSubDel <- with(wgs[!grepl("790|851", subject)],
                       evalPred(edDel, wgDel))
predMetrics <- as.data.table(do.call(rbind, pmLst), keep.rownames = "PredSet")
predMetrics
```

```
##      PredSet   MCC   TPR   FDR    PPV BalAcc
##  1:       mc 0.185 0.335 0.897 0.1030  0.666
##  2:       ed 0.263 0.363 0.809 0.1910  0.681
##  3:    mcSub 0.487 0.345 0.311 0.6890  0.672
##  4:    edSub 0.482 0.378 0.383 0.6170  0.689
##  5:    mcDup 0.396 0.236 0.334 0.6660  0.618
##  6:    edDup 0.347 0.240 0.496 0.5040  0.620
##  7: mcSubDup 0.404 0.246 0.333 0.6670  0.623
##  8: edSubDup 0.384 0.266 0.446 0.5540  0.633
##  9:    mcDel 0.180 0.639 0.949 0.0509  0.818
## 10:    edDel 0.219 0.558 0.914 0.0861  0.778
## 11: mcSubDel 0.683 0.661 0.294 0.7060  0.830
## 12: edSubDel 0.541 0.554 0.471 0.5290  0.777
```

```
ctsAll <- euler(wgs[ , .(mc, ed, wg)])
ctsSub <- euler(wgs[!grepl("790|851", subject), .(mc, ed, wg)])
ctsAllDup <- euler(wgs[ , .(mcDup, edDup, wgDup)])
ctsSubDup <- euler(wgs[!grepl("790|851", subject), .(mcDup, edDup, wgDup)])
ctsAllDel <- euler(wgs[ , .(mcDel, edDel, wgDel)])
ctsSubDel <- euler(wgs[!grepl("790|851", subject), .(mcDel, edDel, wgDel)])
eulerr_options(fills = list(fill = c("#E9E9E9", "#7F7FC4", "#FFC57F")),
               quantities = list(cex = 0.5))
gridFigLab <- function(lab) {
  grid.text(lab, x = 0, y = 1, hjust = 0, vjust = 1, gp = gpar(font = 2))
}
plot(ctsSubDup, quantities = TRUE, labels = FALSE, main = "")
gridFigLab("A")
grid.text("DUPLICATIONS", x = 0.5, y = 0.9)
plot(ctsSubDel, quantities = TRUE, labels = FALSE, main = "")
gridFigLab("B")
grid.text("DELETIONS", x = 0.5, y = 0.9)
plot(ctsAllDup, quantities = TRUE, labels = FALSE, main = "")
gridFigLab("C")
grid.text("DUPLICATIONS", x = 0.5, y = 0.9)
plot(ctsAllDel, quantities = TRUE, labels = FALSE, main = "")
gridFigLab("D")
grid.text("DELETIONS", x = 0.5, y = 0.9)
```

```
par(mar = rep(0, 4))
plot.new()
legend(x = "center",
       legend = c("MC", "ED", "WG"),
       fill = c("#E9E9E9", "#7F7FC4", "#FFC57F"),
       horiz = TRUE,
       bty = "n")
```

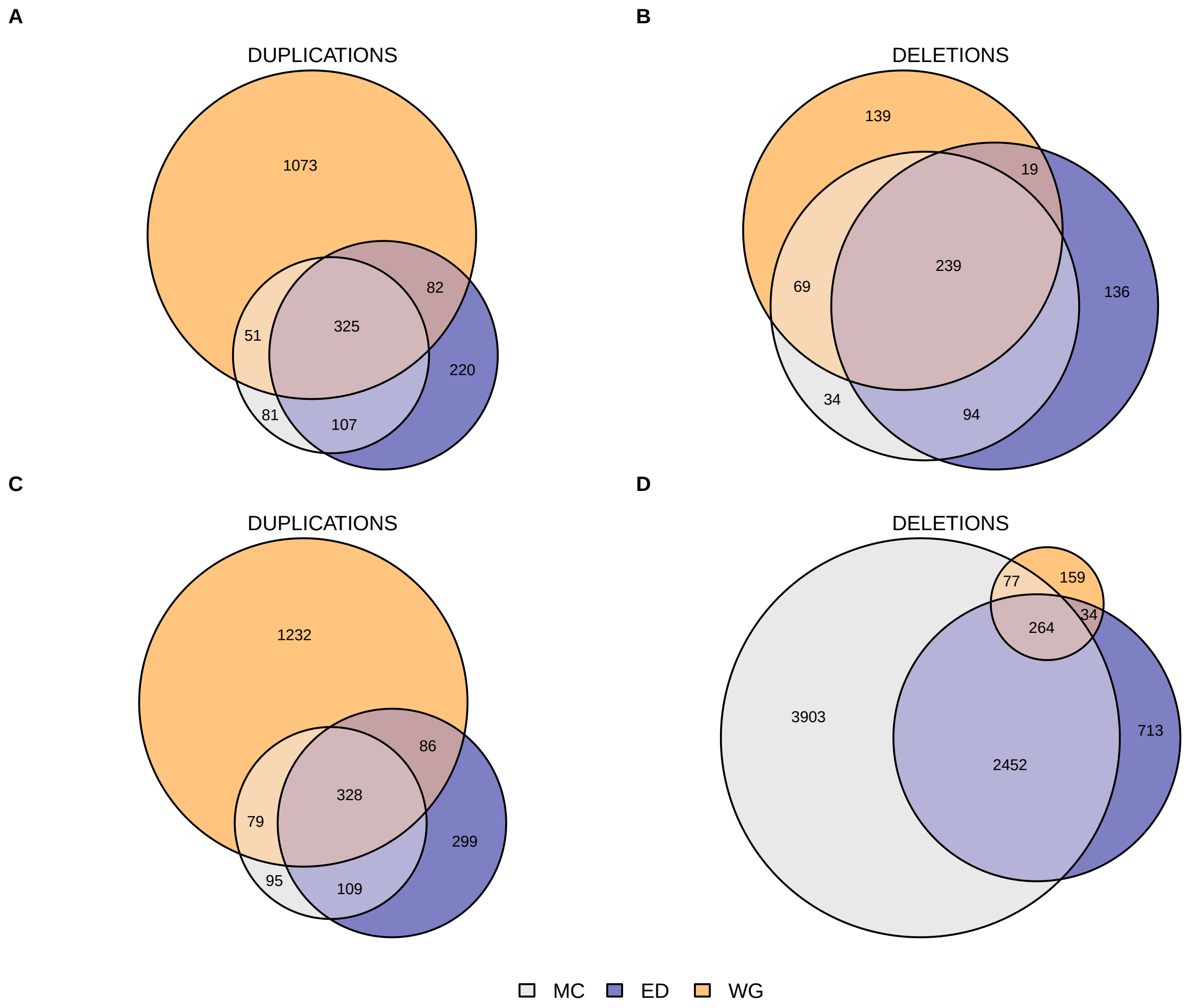

Figure S6: (A-B) included in manuscript; (C-D) show the concordance of duplications and deletions for the whole WGS pool. mcCNV calls in gray; ExomeDepth calls in blue; ERDS/cnvpytor calls in orange.

7 Slurm scripts to perform simulation study

7.1 Create count objects

```
##---------------------------------------------------------------------------##
## Script to create the varDepth simulated counts
##---------------------------------------------------------------------------##

library(mcCNV)
library(rslurm)
library(data.table)
library(filer2020A)
```

```r
## Directory for storing the data
wd <- getwd()

data(subjectMeta)
pl1Int <- subsetCounts(subjectMeta[pool == "Pool1", subject])
pl1Int <- pl1Int[ , .(ttl = sum(molCount)), by = .(seqnames, start, end)]
pl1Int[ , captureProb := ttl/sum(ttl)]
pl1Int[ , ttl := NULL]

deps <- as.integer(seq(5, 100, 5)) ## Sequencing depths

## Set up the file system
odir <- file.path(wd, "varDepthCounts")
if (dir.exists(odir)) unlink(odir, recursive = TRUE, force = TRUE)
dir.create(odir)

pars <- data.table(expand.grid(dep = deps, rep = seq(200)))
set.seed(1234)
pars[ , seed := sample(1e6, .N)]
pars[ , odir := odir]

simPool <- function(dep, rep, seed, odir) {
  wndw <- as.integer(c(dep - 0.3*dep, dep + 0.3*dep)*1e6)
  cnt <- try(cnvSimPool(nSubjects = 16L,
                        countRange = wndw,
                        interval = pl1Int,
                        seed = seed,
                        variantWidth = 1L))
  fname <- sprintf("varDepth_d%0.3d_r%0.4d.counts", dep, rep)
  saveRDS(cnt, file = file.path(odir, fname))
  !is(cnt, 'try-error')
}

slurm_apply(f = simPool,
            add_objects = "pl1Int",
            params = pars,
            nodes = nrow(pars),
            cpus_per_node = 1,
            jobname = "varDepthCounts",
            slurm_options = list(mem = 8000,
                                 array = sprintf("0-%d%%%d",
                                                 nrow(pars) - 1,
                                                 1000),
                                 'cpus-per-task' = 1,
                                 error =  "%A_%a.err",
                                 output = "%A_%a.out",
                                 time = "2-00:00:00"))
```

7.2 Run mcCNV on count objects

```
##----------------------------------------------------------------------------##
## Script to perform mcCNV algorithm on varDepth simulation
##----------------------------------------------------------------------------##

library(mcCNV)
library(rslurm)
library(data.table)
library(stringr)
library(filer2020A)

## Directory for storing the data
wd <- getwd()

pars <- data.table(fl = Sys.glob(file.path(wd, "varDepthCounts/*.counts")))
pars[ , dep := as.integer(sub("d", "", str_extract(fl, "d[0-9]{3}")))]
## Set up the file system
odir <- file.path(wd, "varDepthMcCalls")
if (dir.exists(odir)) unlink(odir, recursive = TRUE, force = TRUE)
dir.create(odir)

pars[ , odir := odir]

doCalc <- function(fl, dep, odir) {
  dat <- readRDS(fl)
  ## Setup output
  ifl <- basename(fl)
  rep <- as.integer(sub("r", "", str_extract(ifl, "r[0-9]{4}")))
  ofmt <- sub(".counts$", ".mcCallSummary", ifl)
  mcFl <- file.path(odir, ofmt)
  ## mcCNV calls
  mcCalls <- try(cnvCallCN(counts = dat, verbose = TRUE))
  mcFail <- is(mcCalls, 'try-error')
  if (!mcFail) {
    mcRes <- mcCalls[ , .N, by = .(actCN, CN, passFilter)]
    mcRes[ , dep := dep]
    mcRes[ , rep := rep]
    saveRDS(mcRes, file = mcFl)
  }
  !mcFail
}

slurm_apply(f = doCalc,
            params = pars,
            nodes = nrow(pars),
            cpus_per_node = 1,
            jobname = "varDepthMcCalls",
            slurm_options = list(mem = 12000,
                                 array = sprintf("0-%d%%%d",
                                                 nrow(pars) - 1,
                                                 1000),
                                 'cpus-per-task' = 1,
```

```
                                  error =  "%A_%a.err",
                                  output = "%A_%a.out",
                                  time = "10-00:00:00"))
```

7.3 Run ExomeDepth with default parameters on count objects

```
##------------------------------------------------------------------------------##
## Script to perform ExomeDepth algorithm w/ defaults on varDepth simulation
##------------------------------------------------------------------------------##

library(mcCNV)
library(rslurm)
library(data.table)
library(stringr)
library(filer2020A)

## Directory for storing the data
wd <- getwd()

pars <- data.table(fl = Sys.glob(file.path(wd, "varDepthCounts/*.counts")))
pars[ , dep := as.integer(sub("d", "", str_extract(fl, "d[0-9]{3}")))]
## Set up the file system
odir <- file.path(wd, "varDepthEdDefaultCalls")
if (dir.exists(odir)) unlink(odir, recursive = TRUE, force = TRUE)
dir.create(odir)

pars[ , odir := odir]

doCalc <- function(fl, dep, odir) {
  dat <- readRDS(fl)
  ## Setup output
  ifl <- basename(fl)
  rep <- as.integer(sub("r", "", str_extract(ifl, "r[0-9]{4}")))
  ofmt <- sub(".counts$", ".edDefaultCallSummary", ifl)
  edFl <- file.path(odir, ofmt)
  ## ExomeDepth calls
  edCalls <- try(runExomeDepth(counts = dat))
  edFail <- is(edCalls, 'try-error')
  if (!edFail) {
    setkey(edCalls$calls, subject, seqnames, start, end)
    setkey(dat, subject, seqnames, start, end)
    edRes <- edCalls$calls[dat][ , .N, by = .(actCN, type)]
    edRes[ , dep := dep]
    edRes[ , rep := rep]
    saveRDS(edRes, file = edFl)
  }
  !edFail
}
```

```r
slurm_apply(f = doCalc,
            params = pars,
            nodes = nrow(pars),
            cpus_per_node = 1,
            jobname = "varDepthEdDefaultCalls",
            slurm_options = list(mem = 12000,
                                 array = sprintf("0-%d%%%d",
                                                 nrow(pars) - 1,
                                                 1000),
                                 'cpus-per-task' = 1,
                                 error =  "%A_%a.err",
                                 output = "%A_%a.out",
                                 time = "10-00:00:00"))
```

7.4 Run ExomeDepth with simulation-matched parameters on count objects

```r
##----------------------------------------------------------------------------##
## Script to perform ExomeDepth algorithm w/ best pars on varDepth simulation
##----------------------------------------------------------------------------##

library(mcCNV)
library(rslurm)
library(data.table)
library(stringr)
library(filer2020A)

## Directory for storing the data
wd <- getwd()

pars <- data.table(fl = Sys.glob(file.path(wd, "varDepthCounts/*.counts")))
pars[ , dep := as.integer(sub("d", "", str_extract(fl, "d[0-9]{3}")))]
## Set up the file system
odir <- file.path(wd, "varDepthEdBestCalls")
if (dir.exists(odir)) unlink(odir, recursive = TRUE, force = TRUE)
dir.create(odir)

pars[ , odir := odir]

doCalc <- function(fl, dep, odir) {
  dat <- readRDS(fl)
  ## Setup output
  ifl <- basename(fl)
  rep <- as.integer(sub("r", "", str_extract(ifl, "r[0-9]{4}")))
  ofmt <- sub(".counts$", ".edBestCallSummary", ifl)
  edFl <- file.path(odir, ofmt)
  ## ExomeDepth calls
  edCalls <- try(runExomeDepth(counts = dat,
                               transProb = 0.001,
                               cnvLength = 1000))
```

```
  edFail <- is(edCalls, 'try-error')
  if (!edFail) {
    setkey(edCalls$calls, subject, seqnames, start, end)
    setkey(dat, subject, seqnames, start, end)
    edRes <- edCalls$calls[dat][ , .N, by = .(actCN, type)]
    edRes[ , dep := dep]
    edRes[ , rep := rep]
    saveRDS(edRes, file = edFl)
  }
  !edFail
}

slurm_apply(f = doCalc,
            params = pars,
            nodes = nrow(pars),
            cpus_per_node = 1,
            jobname = "varDepthEdBestCalls",
            slurm_options = list(mem = 12000,
                                 array = sprintf("0-%d%%%d",
                                                 nrow(pars) - 1,
                                                 1000),
                                 'cpus-per-task' = 1,
                                 error =  "%A_%a.err",
                                 output = "%A_%a.out",
                                 time = "10-00:00:00"))
```

8 Snakemake notes

For the Snakemake files to run, the files must be organized into a specific directory structure. Within the exome analysis directory, pools are identified by the following structure:

```
poolName/inputs/sampleName/laneID/R1.fastq.gz
poolName/inputs/sampleName/laneID/R2.fastq.gz
```

Runs within the same 'sampleName' will be merged into a single BAM file. For example, the following shows the files for the NCG_00790 sample within the WGS pool.

```
# WGS/inputs/NCG_00790/
# ├── 190522_UNC41-A00434_0034_AHKL7YDSXX-GATGAATC_S6_L001
#     ├── NCG_00790-CNV_Exome_XT2_GATGAATC_S6_L001_R1_001.fastq.gz
#     └── NCG_00790-CNV_Exome_XT2_GATGAATC_S6_L001_R2_001.fastq.gz
# ├── 190522_UNC41-A00434_0034_AHKL7YDSXX-GATGAATC_S6_L002
#     ├── NCG_00790-CNV_Exome_XT2_GATGAATC_S6_L002_R1_001.fastq.gz
#     └── NCG_00790-CNV_Exome_XT2_GATGAATC_S6_L002_R2_001.fastq.gz
# ├── 190522_UNC41-A00434_0034_AHKL7YDSXX-GATGAATC_S6_L003
#     ├── NCG_00790-CNV_Exome_XT2_GATGAATC_S6_L003_R1_001.fastq.gz
#     └── NCG_00790-CNV_Exome_XT2_GATGAATC_S6_L003_R2_001.fastq.gz
```

```
# ├── 190830_UNC41-A00434_0050_AHCLVLDRXX-GATGAATC_S6_L001
#     ├── NCG_00790-CNV_Exome_XT2_GATGAATC_S6_L001_R1_001.fastq.gz
#     └── NCG_00790-CNV_Exome_XT2_GATGAATC_S6_L001_R2_001.fastq.gz
# └── 190830_UNC41-A00434_0050_AHCLVLDRXX-GATGAATC_S6_L002
#     ├── NCG_00790-CNV_Exome_XT2_GATGAATC_S6_L002_R1_001.fastq.gz
#     └── NCG_00790-CNV_Exome_XT2_GATGAATC_S6_L002_R2_001.fastq.gz
```

The genome file follows the same convention, but simply requires `inputs` and samples are not subdivided by pool.

The provided config and cluster files will need to be updated to match the cluster envrionment used; they are provided as were used as a guide. The following shows a paired-down directory structure for the exome analysis; note the symlink to 'target.intervals' within the pool folder. The 'target.intervals' file should point to a .RDS object with a valid mcCNV interval object (see `?mcCNV::cnvValidInterval`).

```
# wesAnalysis/
# ├── cluster.json
# ├── config.yaml
# ├── envs
#     └── mcCNV.yaml
# ├── runme.snakemake
# ├── scratch
# ├── scripts
#     ├── aggCalls.R
#     ├── callCN.R
#     ├── exomeDepth.R
#     └── getCounts.R
# ├── Snakefile
# ├── slurmOut
# └── WGS
#     ├── inputs
#     └── target.intervals -> path to interval .RDS object
```

For the genome analysis, ERDS will have to be installed manually prior to running the pipeline. We used the following shell script, but have zero expecations it will work within all environments.

```
#!/bin/sh
TOOLDIR=$(pwd)/tools
BINDIR=${TOOLDIR}/bin
ERDS=7f47b69943e254e5ff5a21c7ab8915e9a4da568a

mkdir -p ${BINDIR}

wget "https://github.com/igm-team/ERDS/archive/${ERDS}.zip" -O "${TOOLDIR}/ERDS-${ERDS}.zip"
unzip ${TOOLDIR}/ERDS-${ERDS}.zip -d ${TOOLDIR}

pushd ${TOOLDIR}/ERDS-${ERDS}/erds_tcag/src/phmm; make; popd
```

```
pushd ${TOOLDIR}/ERDS-${ERDS}/erds_tcag/src/hmm; make; popd
pushd ${TOOLDIR}/ERDS-${ERDS}/erds_tcag/src; make; popd

cat <<EOF >${BINDIR}/erds_pipeline
#!/bin/bash
set -eu -o pipefail
export LC_ALL=en_US.UTF-8
perl ${TOOLDIR}/ERDS-${ERDS}/erds_tcag/src/erds_pipeline.pl \$@
EOF

chmod +x ${BINDIR}/erds_pipeline
```

9 Snakemake file for exome anlaysis

Note, Snakemake file and all accessory files contained within the filer2020A package 'inst' directory.

```
configfile: "config.yaml"

GLOB = glob_wildcards('{proj}/inputs/{sample}/{runId}/{fastqBase}.fastq.gz')
SR = dict()
for (p,s,r,f) in zip(*GLOB):
   SR.setdefault(p, {}).setdefault(s, {}).setdefault(r, []).append(f)

rule all:
    input:
        ["%s/fastqc/%s/%s/%s_fastq.zip"%row for row in zip(*GLOB)],
        ["%s/markdup/%s.markdup.bam.flagstat"%row for row in zip(*GLOB[0:2])],
        ["%s/counts/%s.counts"%row for row in zip(*GLOB[0:2])],
        ["%s/markdup/%s.markdup.bam.summary.txt"%row for row in zip(*GLOB[0:2])],
        ["%s/%s.copynumber"%row for row in zip(SR.keys(), SR.keys())],
        ["%s/%s.exomeDepth"%row for row in zip(SR.keys(), SR.keys())],



rule exomeDepth:
    input:
        lambda wildcards:
            expand('{proj}/counts/{sample}.counts',
                   proj = wildcards.proj,
                   sample = SR[wildcards.proj].keys()),
    output: "{proj}/{proj}.exomeDepth"
    log: "{proj}/logs/{proj}.exomeDepth.log"
    conda: "envs/mcCNV.yaml"
    shell:
        "{config[exomeDepth]} --transProb=0.0001 --cnvLength=50000 "
        "--outfile={output} {input} &> {log}"

rule callCN:
    input:
        lambda wildcards:
```

```
            expand('{proj}/counts/{sample}.counts',
                   proj = wildcards.proj,
                   sample = SR[wildcards.proj].keys()),
    output: "{proj}/{proj}.copynumber"
    log: "{proj}/logs/{proj}.copynumber.log"
    conda: "envs/mcCNV.yaml"
    shell:
        "{config[callCN]} --prior=0.2 --width=1 "
        "--outfile={output} {input} &> {log}"

rule getcounts:
    input:
        bamfile = "{proj}/markdup/{sample}.markdup.bam",
        intfile = "{proj}/target.intervals"
    output: "{proj}/counts/{sample}.counts"
    log: "{proj}/logs/counts/{sample}.counts.log"
    conda: "envs/mcCNV.yaml"
    shell:
        "{config[getCounts]} {input.bamfile} {input.intfile} {output} &> {log}"

rule flagstat:
    input: "{proj}/markdup/{sample}.markdup.bam"
    output: "{proj}/markdup/{sample}.markdup.bam.flagstat"
    wrapper: "0.61.0/bio/samtools/flagstat"

rule alignmetrics:
    input:
        bam = "{proj}/markdup/{sample}.markdup.bam",
        ref = config['bwa_index']
    output: "{proj}/markdup/{sample}.markdup.bam.summary.txt"
    log: "{proj}/logs/picard/collectalignmentsummarymetrics/{sample}.log"
    wrapper: "0.61.0/bio/picard/collectalignmentsummarymetrics"

rule markdup:
    input: "{proj}/merged/{sample}.bam"
    output:
        bam = "{proj}/markdup/{sample}.markdup.bam",
        bai = "{proj}/markdup/{sample}.markdup.bai",
        metrics = "{proj}/markdup/{sample}.markdup.metrics.txt"
    log: "{proj}/logs/picard/markdup/{sample}.log"
    params:
        "REMOVE_DUPLICATES=false",
        "CREATE_INDEX=true",
        'TMP_DIR="%s"'%config['temp_dir']
    wrapper: "0.61.0/bio/picard/markduplicates"

rule merge:
    input:
        lambda wildcards:
            expand('{proj}/mapped/{sample}.{runId}.bam',
                   proj = wildcards.proj,
```

```
                    sample = wildcards.sample,
                    runId = SR[wildcards.proj][wildcards.sample])
    output: bam = temp("{proj}/merged/{sample}.bam")
    log: "{proj}/logs/picard/merged/{sample}.log"
    params: "VALIDATION_STRINGENCY=LENIENT"
    wrapper: "0.61.0/bio/picard/mergesamfiles"

rule bwamem:
    input:
        reads = lambda wildcards:
            expand('{proj}/inputs/{sample}/{runId}/{fastq}.fastq.gz',
                    proj = wildcards.proj,
                    sample = wildcards.sample,
                    runId = wildcards.runId,
                    fastq = SR[wildcards.proj][wildcards.sample][wildcards.runId])
    output: temp("{proj}/mapped/{sample}.{runId}.bam")
    log: "{proj}/logs/bwa_mem/{sample}.{runId}.log"
    params:
        index = config['bwa_index'],
        extra = r"-R '@RG\tID:{sample}_{runId}\tSM:{sample}\tPL:Illumina\tCN:UNC'",
        sort = "picard",
        sort_order = "coordinate",
        sort_extra = 'TMP_DIR="%s"'%config['temp_dir']
    threads: 8
    wrapper: "0.61.0/bio/bwa/mem"

rule fastqc:
    input: "{proj}/inputs/{sample}/{runId}/{stem}.fastq.gz",
    output:
        html = "{proj}/fastqc/{sample}/{runId}/{stem}.html",
        zip = "{proj}/fastqc/{sample}/{runId}/{stem}_fastq.zip"
    params: ""
    wrapper: "0.61.0/bio/fastqc"
```

10 Snakemake file for genome anlaysis

Note, Snakemake file and all accessory files contained within the filer2020A package 'inst' directory.

```
configfile: "config.yaml"

sampleRuns = dict()
fastqs = []
flagstats = []
alignstats = []
calls = []
deps = []
cnvpytor = []
erds = []
combine = []
```

```
for (s,r,f) in zip(*glob_wildcards('inputs/{sample}/{runId}/{fastqBase}.fastq.gz')):
    sampleRuns.setdefault(s, {}).setdefault(r, []).append(f)
    fastqs.append('fastqc/%s/%s/%s_fastqc.zip'%(s,r,f))
    flagstats.append('sorted/%s.sorted.markdup.bam.flagstat'%(s))
    alignstats.append('sorted/%s.sorted.markdup.bam.alignMetrics'%(s))
    calls.append('calls/%s.g.vcf'%(s))
    deps.append('depths/%s.depth.quantile'%(s))
    cnvpytor.append('cnvpytor/%s.pytor.calls'%(s))
    erds.append('erds/%s/%s.events'%(s,s))
    combine.append('combined/%s.cnvcalls'%(s))

rule all:
    input:
        fastqs,
        flagstats,
        alignstats,
        calls,
        deps,
        cnvpytor,
        erds,
        combine,

rule fastqc:
    input:
        "inputs/{sample}/{runId}/{stem}.fastq.gz"
    output:
        html="fastqc/{sample}/{runId}/{stem}.html",
        zip="fastqc/{sample}/{runId}/{stem}_fastqc.zip"
    params: ""
    wrapper:
        "0.49.0/bio/fastqc"

rule bwamem:
    input:
        reads=lambda wildcards:
            expand('inputs/{sample}/{runId}/{stem}.fastq.gz',
                   sample=wildcards.sample,
                   runId=wildcards.runId,
                   stem=sampleRuns[wildcards.sample][wildcards.runId])
    output:
        temp("mapped/{sample}.{runId}.bam")
    log:
        "logs/bwa_mem/{sample}.{runId}.log"
    params:
        index=config['bwa_index'],
        extra=r"-R '@RG\tID:{sample}_{runId}\tSM:{sample}\tPL:Illumina\tCN:UNC'",
        sort="picard",
        sort_order="queryname",
        sort_extra='TMP_DIR="%s"'%config['temp_dir']
    threads: 8
    wrapper:
```

```
        "0.49.0/bio/bwa/mem"

rule merge:
    input: lambda wildcards:
            expand('mapped/{sample}.{runId}.bam',
                   sample=wildcards.sample,
                   runId=sampleRuns[wildcards.sample])
    output: temp("merged/{sample}.bam")
    params: "-n"
    threads: 8
    wrapper:
        "0.49.0/bio/samtools/merge"

rule markdup:
    input:
        "merged/{sample}.bam"
    output:
        bam = temp("markdup/{sample}.markdup.bam"),
        metrics = "markdup/{sample}.markdup.markdupMetrics"
    log:
        "logs/picard/markdup/{sample}.log"
    params:
        "REMOVE_DUPLICATES=false",
        "ASSUME_SORT_ORDER=queryname",
        "CREATE_INDEX=false",
        'TMP_DIR="%s"'%config['temp_dir']
    wrapper:
        "0.49.0/bio/picard/markduplicates"

rule sort:
    input: "markdup/{sample}.markdup.bam"
    output: "sorted/{sample}.sorted.markdup.bam"
    params: "-m 2G"
    threads: 8
    wrapper:
        "0.49.0/bio/samtools/sort"

rule index:
    input: "sorted/{sample}.sorted.markdup.bam"
    output: "sorted/{sample}.sorted.markdup.bam.bai"
    wrapper:
        "0.49.0/bio/samtools/index"

rule stats:
    input:
        bam = "sorted/{sample}.sorted.markdup.bam",
        ref = config['bwa_index'],
idx = "sorted/{sample}.sorted.markdup.bam.bai"
    output:
        "sorted/{sample}.sorted.markdup.bam.alignMetrics"
    log:
```

```
        "logs/picard/collectalignmentsummarymetrics/{sample}.log"
    wrapper:
        "0.49.0/bio/picard/collectalignmentsummarymetrics"

rule samtools_depth:
    input:
        bams="sorted/{sample}.sorted.markdup.bam",
    output: "depths/{sample}.depth"
    params:
        # optional bed file passed to -b
        extra="" # optional additional parameters as string
    wrapper:
        "0.60.0/bio/samtools/depth"

rule depthQuantile:
    input: "depths/{sample}.depth"
    output: "depths/{sample}.depth.quantile"
    conda: "envs/R.yaml"
    shell: "{config[depthQuantile]} {input} {output}"

rule samtools_flagstat:
    input: "sorted/{sample}.sorted.markdup.bam"
    output: "sorted/{sample}.sorted.markdup.bam.flagstat"
    wrapper:
        "0.49.0/bio/samtools/flagstat"

rule gatkBqsr:
    input:
        bam = "sorted/{sample}.sorted.markdup.bam",
        ref = config['bwa_index'],
        known = config['dbsnp']
    output:
        bam = "recal/{sample}.recal.bam"
    log:
        "logs/gatk/bqsr/{sample}.log"
    params:
        extra = "",  # optional
        java_opts = "-Xmx20G -XX:ParallelGCThreads=8", # optional
    threads: 8
    wrapper:
        "0.49.0/bio/gatk/baserecalibrator"

rule haplotypeCaller:
    input:
        # single or list of bam files
        bam = "recal/{sample}.recal.bam",
        ref = config['bwa_index']
    output:
        gvcf = "calls/{sample}.g.vcf",
    log:
        "logs/gatk/haplotypecaller/{sample}.log"
```

```
    params:
        extra = "",  # optional
        java_opts = "-Xmx20G -XX:ParallelGCThreads=8", # optional
    threads: 8
    wrapper:
        "0.49.0/bio/gatk/haplotypecaller"

rule cnvpytor:
    input:
        bam = "recal/{sample}.recal.bam",
        ref = config['bwa_index']
    output:
        pytor = "cnvpytor/{sample}.pytor",
        calls = "cnvpytor/{sample}.pytor.calls"
    params:
        binsize = 500
    shell:
        """
        cnvpytor -root {output.pytor} -rd {input.bam} -T {input.ref}
        cnvpytor -root {output.pytor} -his {params.binsize}
        cnvpytor -root {output.pytor} -partition {params.binsize}
        cnvpytor -root {output.pytor} -call {params.binsize} > {output.calls}
        """

rule erds:
    input:
        bam = "recal/{sample}.recal.bam",
        ref = config['bwa_index'],
        vcf = "calls/{sample}.g.vcf",
    output: "erds/{sample}/{sample}.events"
    params:
        dr = "erds/{sample}"
    conda: "envs/erds.yaml"
    shell:
        "{config[erds]} -o {params.dr} -b {input.bam} "
        "-v {input.vcf} -r {input.ref}"

rule combineCalls:
    input:
        pytor = "cnvpytor/{sample}.pytor.calls",
        erds = "erds/{sample}/{sample}.events",
        interval = "targets.intervals",
    output: "combined/{sample}.cnvcalls"
    conda: "envs/R.yaml"
    shell: "{config[combineCalls]} {wildcards.sample} {input.pytor} {input.erds} "
           "{input.interval} {output}"
```
